# Competencies supporting high-performance translational teams: A review of the SciTS evidence base

**DOI:** 10.1017/cts.2023.17

**Published:** 2023-03-03

**Authors:** Allan R. Brasier, Elizabeth S. Burnside, Betsy Rolland

**Affiliations:** 1 Institute for Clinical and Translational Research, School of Medicine and Public Health, University of Wisconsin-Madison, Madison, WI, USA; 2 Carbone Cancer Center, School of Medicine and Public Health, University of Wisconsin-Madison, Madison, WI, USA

**Keywords:** Competencies, team science, knowledge skills attributes, translational teams, team training

## Abstract

A translational team (TT) is a specific type of interdisciplinary team that seeks to improve human health. Because high-performing TTs are critical to accomplishing CTSA goals, a greater understanding of how to promote TT performance is needed. Previous work by a CTSA Workgroup formulated a taxonomy of 5 interrelated team-emergent competency “domains” for successful translation: 1). affect, 2). communication, 3). management, 4). collaborative problem-solving, and 5). leadership. These Knowledge Skills and Attitudes (KSAs) develop within teams from the team’s interactions. However, understanding how practice in these domains enhance team performance was unaddressed. To fill this gap, we conducted a scoping literature review of empirical team studies from the broader Science of Team Science literature domains. We identified specific team-emergent KSAs that enhance TT performance, mapped these to the earlier “domain” taxonomy, and developed a rubric for their assessment. This work identifies important areas of intersection of practices in specific competencies across other competency domains. We find that inclusive environment, openness to transdisciplinary knowledge sharing, and situational leadership are a core triad of team-emergent competencies that reinforce each other and are highly linked to team performance. Finally, we identify strategies for enhancing these competencies. This work provides a grounded approach for training interventions in the CTSA context.

## Introduction

**Fig. 1.**
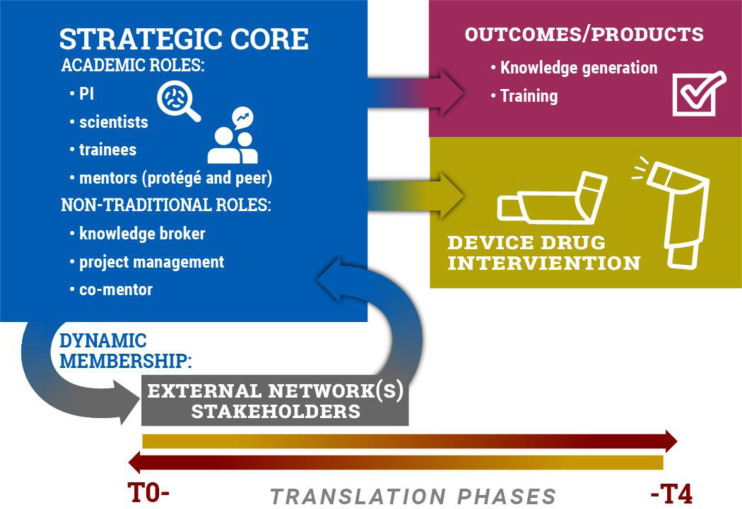
The translational team (TT) model. A schematic of the strategic core of a CTSA-type TT. The strategic core includes the personnel involved in the translational research across its lifespan, whose integration and effective interactions are essential for team success. These members include traditional academic roles [such as the principal investigator, early career trainee (e.g., a CTSA-funded KL2 scholar), research scientists] and those in nontraditional roles (knowledge brokers, project managers, and mentors). During the conduct of translational research, the strategic core interfaces with external scientific and professional networks, including scientific societies, professional societies, and clinical research programs. In addition, external stakeholders (patient advocacy groups, industry partners, community groups) also play important roles at various stages of translation. As the TT advances across the phases of the translational spectrum, from preclinical (T0) to clinical and community adoption (T4), the TT generates two major outcomes. One type of outcomes is knowledge generation and training, characteristics of academic knowledge-generating teams. Another outcome is development of a drug/device/intervention, characteristic of an industry product development team.

A growing body of Science of Team Science (SciTS) research has demonstrated that interdisciplinary and inter-institutional teams produce knowledge and products that are most impactful in the field and result in greater societal benefit [1]. Consequently, the CTSA consortium has embraced the interdisciplinary team approach to advance clinical and translational research to meaningful health outcomes [2]. Unique in its construction, tasks, environment, and dynamic membership, a translational team (TT) is a hybrid of an organizational knowledge-generating team and an industry-like product development team operating within an academic environment that seeks to span T0–-T4 phases of translational research [3]. Specifically, a TT is composed of a diverse, dynamically engaged membership that interacts, adapts, and evolves to advance a product (device/drug/diagnostic) or evidence-based intervention (process or behavioral intervention) toward clinical or community interventions to improve human health (Fig. 1, [3–5]).

Factors promoting TT development and performance are understudied. Observational studies of the lifecycle of 11 TTs composed of over 100 members in the CTSA environment have shown that TTs transition across the translational spectrum, maturing and refining their research plan, conducting interdependent research linked to output (knowledge, manuscripts, grants), and providing societal/clinical impact from translational intervention [5,6]. These TTs were composed of a core nucleus of members including the principal investigator, scientists, and trainees that were relatively constant over the evolution of the translational project. This core nucleus had dynamic participation of scientists with specialized technology skills or patient stakeholders that formed transient interactions with the TT at different stages of its translational pathway (Fig. 1, [5]). Although this observational study helped to inform the major constituents and pathway of the TT, the processes or skills exhibited by the high-performing TTs were not completely developed.

**Fig. 2.**
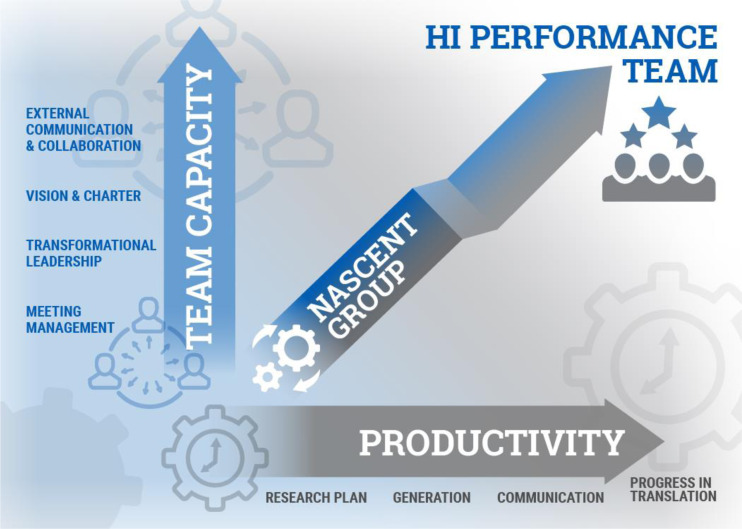
Dimensions of translational team (TT) success. Schematic diagram of the maturation of a TT using an input-output-process perspective. Here, the maturation of a TT from nascent group to high-performance team occurs in two dimensions. Along the *X*-axis, productivity, a translational team advances in terms of outcomes along the translational research spectrum, developing a research plan, generating knowledge, communicating their findings by manuscripts, and progressing its translational product to clinical application. On the *Y*-axis, team capacity, is the growth in team processes that support this maturation. These team processes include, but are not limited to, meeting management, transformational leadership, shared vision, and external collaboration. The angled arrow indicates that the growth in capacity and outcomes are not necessarily at the same rate.

For the purpose of this review, we use the term “performance” to mean advancement across the translational research spectrum [5,6]. Inherent in the popular Input-Process-Outcome model of team development, it is assumed that growth in team processes enhances team performance (Fig. 2). However, the most important KSAs supporting a high-performing TT have not been fully defined. To initially address this question, an expert panel of members from a CTSA Team Science Affinity Workgroup and CTSA-sponsored Domain Task Force used a comprehensive literature review and modified Delphi method to identify individual and team-emergent competency “domains.” This work separated competencies needed by an individual to effectively participate in the TT from those emerging as a consequence of team member interactions [4,7]. These latter team-emergent competency domains are as follows: 1). ***affect***, a domain describing that the bonds between TT members grounded in a concern, empathy, and shared regard for others [8]; 2. ***communication***, a state where the TT effectively exchanges information and integrates team member expertise to solve research problems [9]; 3. ***management***, a term referring to leadership actions that effectively organize and sustain components of multicomponent investigation [10]; 4. ***collaborative problem-solving***, a process where cognitive and social skills of the TT are used to integrate research findings and discipline-grounded interpretations into a cohesive model [11]; and 5. ***leadership***, the process of providing or supporting the cognitive, resource, and affective needs for a TT [12].

Several important conclusions arose from this work. First, team-emergent competencies arise over time from individual antecedents and are modified by inter-team interactions that are not merely individual behaviors of those on the team. For example, the development of positive team ***affect*** is based on trusting relationships that arise from intra-team interactions. Second, exhibiting specific KSAs in one competency domain reinforces behaviors in other domains, such as ***communication*** promotes ***collaborative problem-solving*** as well as ***affect***. Although this analysis was an important step in identifying effective TT behaviors, more work is needed to identify actionable KSAs, linked to performance, that inform team-focused training and evaluation strategies for CTSA-relevant TTs.

**Fig. 3.**
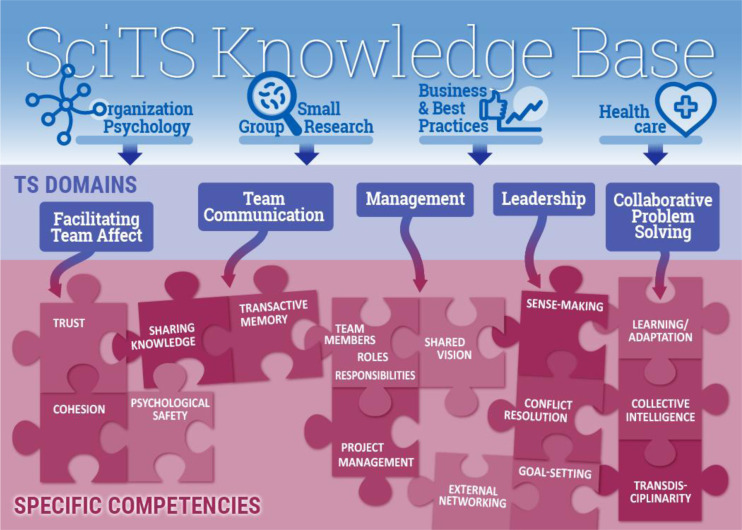
Strategy for competency refinement. Overall strategy for the development of this review. Specific competencies identified in the Science of Team Science (SciTS) Knowledge Base from Organizational Psychology, Small Group Research, Business and Best Practices, and Health Care were identified using literature search. These manuscripts were subjected to a scoping review protocol to identify relevant data for translational team (TT) performance. These studies were mapped to five major translational science (TS) competency domains associated with TT success. For each domain, specific competencies were developed. These 15 specific competencies are most strongly linked to high-performance transdisciplinary teams according to the literature. These are illustrated as puzzle pieces whose assembly and application support high-performance TTs.

An unresolved question is what specific behaviors or skills in these competency domains most directly impact TT performance? To address this important knowledge gap, we conducted a scoping literature review of interdisciplinary studies conducted in the social sciences, education, organizational psychology, business, and medical literature to identify specific KSAs, evaluate those that are most impactful, determine how they may intersect with other domains, and identify how they may be assessed and or improved (Fig. 3). Four hundred and eighty-eight primary articles were identified that included 344 from our primary literature search; these were combined with earlier scoping reviews (*n* = 488) and subjected to a standard scoping review protocol (see Supplementary file S1 for the detailed protocol; illustrative figure and Supplementary file S2 for the full bibliography). From this bibliography, abstracts were evaluated for studies relevant to TTs, competency assessment, and measurement of performance, leading to 162 core manuscripts that were evaluated in detail by the authors and became the core data used in this review (see Supplementary file S3).

The core information is summarized as follows: In Section I, we examine evidence to codify the 15 most important KSAs within the competency domains, describe how these affect TT performance, call out observable behaviors associated with these KSAs, and identify rubrics for assessing them. In Section II, we describe how the practice of select competencies reinforce one another. In Section III, we identify evidence for strategies that have an evidence base demonstrating their potential to enhance KSAs specific to TTs.

## Emergent Competencies Affecting TT Performance

**Fig. 4.**
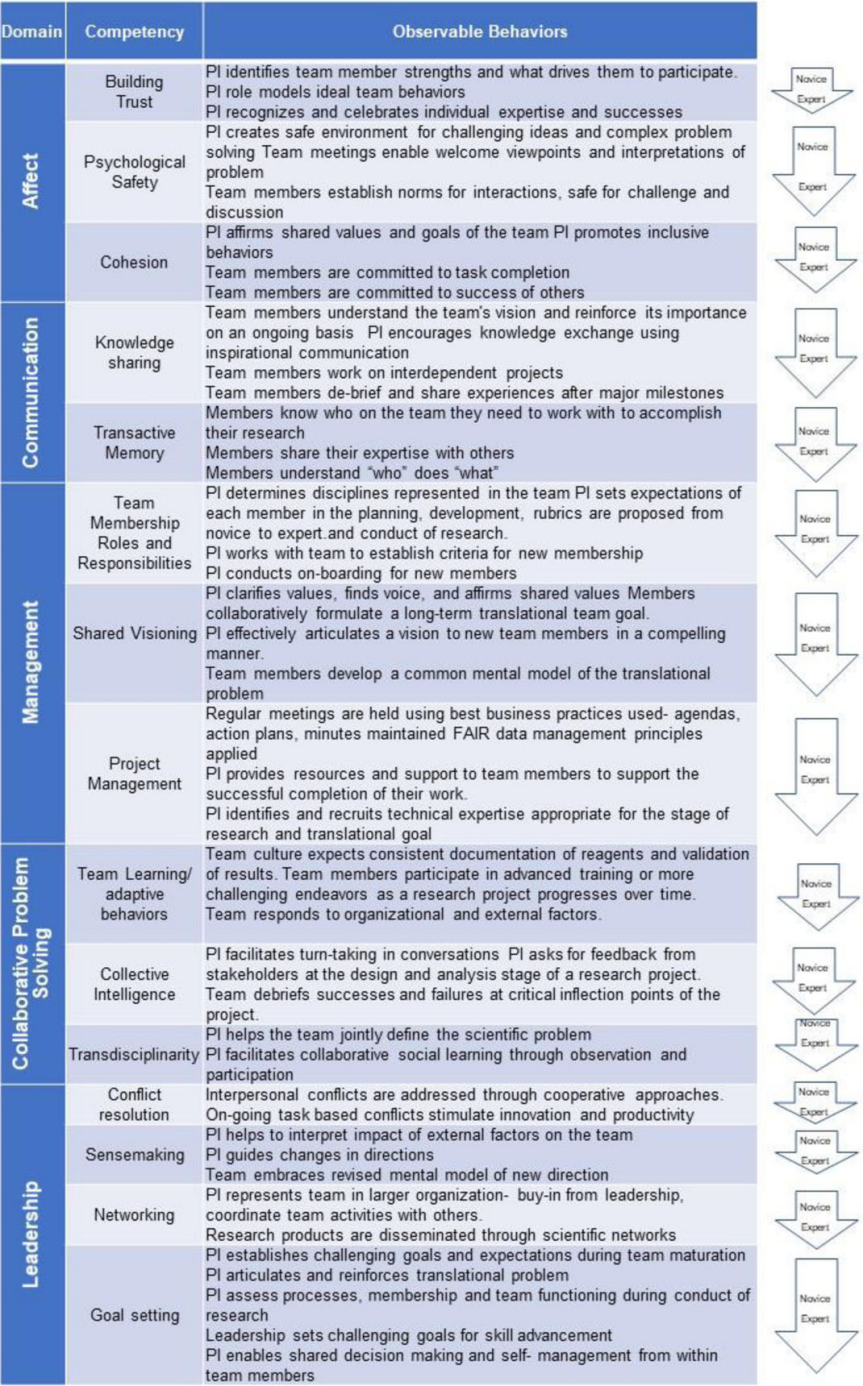
Observable Behaviors of specific competencies within the translational team (TT) competency “domains”. Team-emergent competency domains are shown, grouped by color with the specific competencies for each. For each specific KSA, observable behaviors are listed from simple (novice) to advanced (expert) application. Abbreviations: PI, principal investigator.

Throughout this manuscript, we use the term competencies to signify the KSAs that contribute to team, distinct from individual member, performance [13]. Thus, we often use these terms (competencies and KSAs) interchangeably. Observable behaviors associated with the specific competencies we enumerate below are shown in Fig. 4.

**1. *Affect*** refers to the development of empathy, affiliation, and rapport between members on the basis of shared regard for the other members of the TT [4,8]. We identified that the practice of three complementary specific competencies that mostly advance ***affect*** are *trust, psychological safety,* and *cohesion*. In this setting, *Trust* refers to the confidence that team members have in the abilities of their colleagues to do reproducible work, share results, and discuss their interpretations. Intra-team relationships developed in a trusting environment contribute to *psychological safety*, a shared belief that the team environment is safe for risk-taking, formulating opposing ideas, or challenging team assumptions that plays an important role in highly functioning teams [14]. *Cohesion* refers to the multiple factors that act on members to remain committed to accomplishing the team’s goals [15,16]. Consequently, *cohesion* is complementary, yet distinct from *trust*. Evidence supporting the relationship between these KSAs and team performance, as well as behaviors associated with *trust, psychological safety,* and *cohesion,* are elaborated below:

*Trust* enables a team member to rely on others within the TT because they are accountable, responsible and will support each other when difficulties arise. There is a large body of work that demonstrates a trusting environment has substantial impact on team performance [17,18]. A comprehensive meta-analysis of 112 independent studies (involving 7763 teams) found that intra-team *trust* was one of the strongest predictors of team performance, independent of other associated covariates, including trust in the leader and independent of the team’s past successful performances [19]. Intra-team *trust* is most impactful in teams with high levels of interdependent activities, where projects depend on other team member activities. This interdependency is inherent of complex and innovative research, a characteristic of TT projects. Other studies have shown that developing trust encourages learning, risk-taking, cohesiveness [17,20], knowledge sharing [21], project effectiveness [22], team member satisfaction [23], and creativity [24]. Positive relationships with team leadership support team-emergent resilience, consistency, trust, and competence [25].

A number of team-level characteristics indicate the presence of *trust*. *Trusting* teams are more willing to express ideas and exhibit higher levels of listening, and problem-solving behaviors, leading to the phenomenon of “collective intelligence” [26]. Another manifestation of trusting teams is the enablement of team voice. Team voice is the extent to which team members make constructive suggestions for improvement, share new ideas, and discuss problems or potential problems, or the result of knowledge sharing in a trusting environment [27].

*Psychological safety* is a higher-ordered manifestation of *trust* that refers to the perception of the consequences of taking risks in challenging interpretations, scientific dogma, or team processes [17]. Psychological safety arises from trusting interpersonal relationships developed with other team members, organizational norms, as well as with the team leadership [17]. A large body of work supports that psychological safety is an important component of high-performance teams. For example, educational teams who exhibit a climate of psychological safety use conflicts that arise from taskwork to improve team performance, by developing creative ideas and critical discussion [28]. This finding was replicated in a separate study of 47 business teams where psychological safety was positively related to process innovations and performance [29]. In these ways, building trust is foundational to developing climate of *psychological safety*, contributing in multiple ways to sustained team performance [30].

*Cohesion* refers to the strength and extent of interpersonal connection between team members. *Cohesion* is a multidimensional team attribute that includes task cohesion, social cohesion, and group pride, each linked with team performance in multiple studies [31,32]. Several comprehensive meta-analyses have linked social and task cohesion to performance in teams whose taskwork requires intensive between-member interactions, characteristic of TTs [32]. The relationship between *cohesion* and performance has been consistently positive across many team types, but found to be highly dependent on intra-group processes. *Cohesio*n has been extensively studied in product development teams. Product development teams have many similarities with TTs in that they are composed of cross-functional expertise who develop a specific product over a definitive time period using interdependent activities [3]. An extensive analysis of 157 studies relating task cohesion and social cohesion on performance indicated a positive and strong relationship in project development teams over other team types, such as service teams or production teams [33]. An important characteristic of *cohesion* is this emerges over time, linked with performance, morale, and resiliency [34]. This dynamic property of *cohesion* should be factored into assessment methods, with social and task cohesion being the primary dimensions of importance [15].

**2. *Communication*** is a team-emergent competency domain that refers to the ability to integrate knowledge and expertise in team member interactions and in the task. Team knowledge is the collective understanding of the group on how to coordinate efforts and satisfy needs of other team members. Task knowledge, by contrast, refers to members of a TT having accurate, relevant, and timely information about actions that need to be performed to conduct its research or test a hypothesis. Both forms of communication are critical to effective team performance [35–37]. In this review, we found that *knowledge sharing* is an important specific competency of the ***communication*** domain that supports team knowledge, whereas a *transactive memory system* supports task knowledge.

*Knowledge sharing* is a behavior where team members provide other members with technical information, “know-how” and skills relevant to advancing the team’s translational product. Exchanging knowledge between team members plays a critical role in performance for teams engaging in highly interdependent activities, cultivating innovation/creativity, and navigating complex decision-making processes [38,39]. These factors of interdependence, innovation, and complex decision-making are characteristic of TTs [5]. The evidence that *knowledge sharing* leads to superior team performance comes from studies in new product development teams, R&D teams, and software development teams [40,41], team types that share common processes and interdependencies with TTs [3]. Meta-analytic results from 72 independent studies encompassing over 4795 groups have demonstrated that knowledge sharing leads to improved team performance through enhancement of team interactions and development of shared mental model (SMMs) [42].

*Transactive memory system* is a term that refers to the group-level knowledge of “who” on the team has “what” expertise. A *transactive memory system* is foundational to promoting information sharing and knowledge integration; this knowledge base is developed through reciprocal exchange and joint effort between individual team members engaged in interdependent projects. This knowledge base depends on the extent to which team members get to know one another and establish routines for interaction and task accomplishment [43]. Because of this dependency, a *transactive memory system* develops only as a TT matures during its active research phase. We found that the impact of *transactive memory system* is greatest in technology-focused product development teams, where shared team member knowledge enables teams to deliver products on short timelines. Another important impact of a *transactive memory system* is that this KSA mediates the positive effect of team-focused training, where groups who are trained together outperform those teams whose members are trained individually [44]. As an aside, a study of teams in time-pressured command/control simulations found that acute stress negatively affected the team’s *SMM* and *transactive memory system*, explaining, in part, why team performance may suffer under acute stress [45].

We find that the ***affect*** and ***communication*** competency domains are interdependent. Having trust in teammates is an important foundation upon which team members share knowledge, explore, and contribute to successful task completion; particularly when collaboration is required and when creative solutions are needed for successful outcomes. This interdependence has implications for developing KSAs in team-based training and interventions.

**3. *Management*** is a competency domain, typically exhibited by leadership, that refers to organizing, planning, and executing components of a TT research program. A number of studies have concluded that effective meeting management improves team effectiveness [46,47]. Competence in this domain includes establishing team membership, defining their roles, and managing their interactions.

**Fig. 5.**
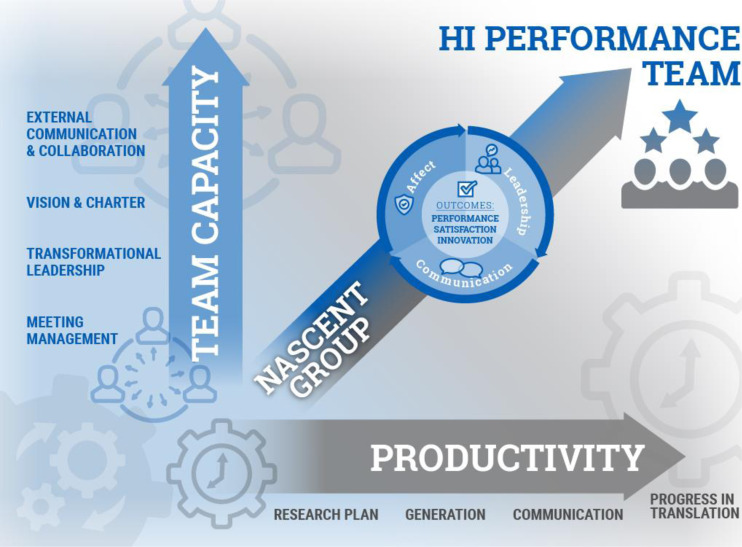
Reinforcing interrelationships of competencies. This analysis suggests that a triad of team-emergent competencies of ***affect, communication,*** and ***leadership*** reinforce and support each other and are highly linked to team performance capacity and productivity. We posit that translational teams displaying these knowledge skills and attitudes at an expert level will advance to high performance.

*Team Membership, Roles, and Responsibilities.* Scientific membership on an interdisciplinary TT is largely influenced by research expertise needed to address current research questions of the TT, and the location of product development along the T0–T4 translational pathway [5]. Noted earlier, the core nucleus of a TT, consisting of the principal investigator, scientists, and KL2 trainees, were relatively constant over the evolution of the translational project (Fig. 1, [5]). In high-performing TTs, the KL2 scholar provided additional leadership skills to complement those of the principal investigators [5]. In addition to providing scientific expertise, the academic stage of collaborating scientists have substantial impact in team productivity. A data-mining study of a large biological sciences department over a 36-year period found that inclusion of postdoctoral fellows account for the large majority of publications, whereas graduate students and postdoctoral fellows with external funding contribute to breakthrough publications [48]. Other studies have found that inclusion of members with prior team experience impacts a team’s ability to learn and adapt to changing environments, affecting team performance [49].

Despite the existence of a core nucleus defined by traditional academic roles (Fig. 1), TTs engage others in its membership, depending on the project needs and the project’s phase in the translational spectrum. In our observational studies, all TTs engaged scientists and members with specific domain expertise – such as technology, informatics, nursing, community, patient groups, trainees, and caregivers at different phases in the translational spectrum [5]. This dynamic affiliation of TT members throughout the different phases of translation has important implications on strategies for onboarding new members by providing “Just-in-Time,” team-focused training approaches.

The definition of member roles is complicated by the findings that team members may play multiple roles throughout the team lifecycle. These include “nontraditional” academic roles, such as knowledge brokers, project managers, role models, and co-mentors. The spectrum and impact of these nontraditional roles on TT performance is understudied. However, current evidence indicates that the role of the knowledge broker is very important. Knowledge brokers are established and connected senior-level scientists that disseminate research discoveries or products throughout the scientific field [50,51]. These dissemination activities enhance the diffusion of knowledge in the scientific field, publication impact, and adoption of new technologies characteristic of successful interdisciplinary teams [1]. In our studies of TTs in the CTSA environment, non-academic team members, such as community or industry members with vested interests in the translational outcome (“stakeholders”), were involved at distinct times as the project matured towards clinical application. Stakeholder involvement brings invaluable perspectives for planning for future dissemination, implementation, and sustainability at the outset of a research effort, known as “Design for Dissemination.” These principles and methods enhance rigorous adoption and sustainable impact of evidence-based innovations of a translational research product [52,53]. The existence and impact of other nontraditional team roles on effective TT will require further study.

Team member diversity (cognitive, gender, race, and ethnicity) impacts team performance in complex ways and is being intensively studied. Cognitive diversity refers to members’ differences in perspective or information processing styles and is not predicted by factors like gender, race, ethnicity, nor age [54]. Cognitive diversity boosts innovation, problem-solving, and collaboration within teams in complex environments [54–57]. Some explanations for this impact are that cognitive diversity increases the connective thinking for solving complex problems [58], enriching the solution space, as well as influencing an emergent collective problem-solving property coined “*collective intelligence*” [26]. Although contrasting ideas and interpretations can lead to innovation and learning in complex situations, a challenge that cognitively diverse teams encounter is that with greater diversity, conflicts can arise. With too much conflict, teams lose clarity of purpose, limiting team function and knowledge integration [59,60]. Consequently, cognitive diversity has an inverted U-shaped relationship with team learning/collective intelligence [56]. Gender diversity has been consistently linked with enhanced team communication, interactive decision-making, and innovation [61]. The literature has shown positive effects of member heterogeneity demographic (race and ethnicity) on team performance, such as creative problem-solving in knowledge-generating teams [62]. An experiment testing racial/ethnic diversity on a specific type of team, a jury, showed that heterogeneous groups took more time to deliberate, had fewer incorrect statements go uncorrected, and performed more accurately [63]. Though the literature is sparse, as with cognitive diversity’s inverted U curve, other types of diversity may lead to dysfunctional team interaction and suboptimal performance [64]. However, it is crucially important to consider any findings related to diversity and performance in the context of the systems in which implicit and explicit bias exists.

Identifying the appropriate membership/roles of a TT is only part of the complexity of effective TT management. Clarifying individual responsibilities that are aligned with the interests and expertise of team members is also vital. With a deep working knowledge of scientific field, the team leader is positioned to assess member skills and align their responsibilities for effective translation. In this process, the leader must ensure that the roles and responsibilities of individual team members are aligned with the overall translational goal and that commitment of resources and credit for participation in team efforts will be shared and assigned. Leader behaviors identifying member characteristics promoting social cohesion [65] and assigning a clear work responsibility are associated with superior team effectiveness [66] and higher team satisfaction [67]. Setting challenging, yet achievable, goals leads to enhanced strategic risk-taking and improves team performance [64,68,69].

*Shared Visioning/mental models.* TTs work toward a collective goal of knowledge generation and bringing an intervention into the clinic or community. This shared, organized understanding and mental representation of knowledge of the team’s goal is also referred to as a *SMM* [70]. *SMMs* are important for team effectiveness, especially when teams are faced with complex, dynamic problems engaging in complex interdependent tasks [71]. It has been proposed that individual characteristics, including prior training, team member longevity, and prior team experience, are antecedents to *SMMs* [72]. Establishing the impact of *SMMs* on interdisciplinary team performance is difficult because this question relies on assessing whether team members share isomorphic cognitive models with others. To address this problem, concept maps have been used to provide evidence that convergence of *SMMs* is associated with team effectiveness [73] by promoting effective team coordination and communication [74]. Of particular relevance to TTs, studies of teams in nonprofit organizations including multiple community stakeholders have found that *SMMs* enable the formation of consistent, collective decisions based on a common understanding of organizational goals and facilitation of stakeholder relationship building [75]. Note above, *SMMs* interface with a *transactive memory system* to enhanced team performance [76,77].

*Project Management.* Meeting management has been well-established to improve effectiveness and sustainability in research teams creating a foundation for effective communication as teams form and build capacity [45,46]. Because project management of interdisciplinary teams is not uniquely specialized for TTs, this topic has been reviewed extensively [78]. Project management can be a strategy for change management [79] and can influence team performance through processes and goal-setting.

More recently, and of special relevance to TTs, work has been focused on dedicated project management as an important role within a TT. The effort devoted to project management within a team depends on the size of its membership and project complexity. For small teams, project management is a nontraditional academic role that may be assumed by a member of the TT core nucleus [80]. By contrast, in complex, multiple institution teams, dedicated consortium directors may play an essential role in team productivity. In the case of these inter-institutional teams, the Consortium Director would be a professional with scientific and administrative knowledge and experience with project coordination, development, data management, and others [81].

**4. *Collaborative problem-solving.*** Collaboration is a complex interaction to provide solution to non-routine problems. Effective collaboration depends on the academic stage of the team members, their prior knowledge, experience in interdependent research projects, and established norms on how the team’s combined knowledge is applied to the translational goal [82]. In an interdisciplinary team, each discipline has its unique worldview – intellectual practices, methods, and biases. ***Collaborative problem-solving*** combines communication with cognitive problem-solving approaches from its team members to result in a shared interpretation or vision [11]. The importance of ***collaborative problem-solving*** as an emergent team behavior has only been recently appreciated [11,83]. Specific team-emergent skills include *learning/adaptation, collective intelligence,* and *transdisciplinarity.*

*Learning/adaptation*. Team interactions are shaped by complex interactions with its environment, often driven by the process of translation itself. The process by which teams respond to changes in their environments that modify their processes is referred to as “adaptation.” Scholars have developed an input-throughput-output model to illustrate the core processes and emergent states underlying team *learning/adaptation* [84-87], resulting in the finding that there is a strong positive relationship between *learning/adaptation* and team performance [88]. Several validated tools for measurement of team adaptability have been developed and empirically tested, including the Job Adaptive Inventory [89]. With this measurement tool, the knowledge base of factors on teams’ adaptability is better understood. From this, we know that individual member flexibility, task expertise, team expertise, and individual adaptability are linked to team *learning/adaptation* [90,91].

*Collective intelligence.* Group collaborative efforts, shared knowledge and skills, and consensus decision-making result in group-level knowledge, coined group, or *collective intelligence.* The development of a statistic that quantifies *collective intelligence,* the “cfactor,” has stimulated research in this phenomenon. From this work, we know that *collective intelligence* is highly predictive of a team’s ability to solve new knowledge tasks; this association has been validated in over 22 studies of 1356 groups [26,92]. *Collective intelligence* is highly correlated with social sensitivity of the group members and turn-taking in conversation.

*Transdisciplinarity.* Of importance to TTs, another important outcome of ***collaborative problem-solving*** is the development of a transdisciplinary research approach [83]. During the initial development of a TT, the participation and scientific problems are “multidisciplinary” with the investigators largely participating from their own discipline [3]. As the TT transcends traditional scientific boundaries to jointly define a problem, conduct problem-solving activity drawing in perspectives from diverse team members, the problem space is broadened to a “transdisciplinary” approach [83,93]. Collaborative learning, an approach that supports cognitive shifts in understanding through observation of and participation with others, is a key component of transdisciplinarity in TTs. Illustrating the reinforcing nature of this team-emergent KSA, ***collaborative problem-solving*** is highly dependent on the strength of ***communication*** (*knowledge sharing*) and **management** (*cognitive diversity*) [94].

***5. Leadership.*** TTs conduct repeated cycles of experimentation, analysis, hypothesis refinement within a complex social and organizational environment. ***Leadership*** seeks to satisfy a team’s needs by providing the cognitive, motivational, affective, and management processes to help the team thrive in that complex environment [12]. Team leaders provide essential support throughout the lifecycle of a TT, including establishing membership, defining roles, setting expectations, providing feedback, and promoting an environment of psychological safety, teamwork/adaptation, discussed above. During the initial formation of the TT, leadership is primarily provided by the principal investigator. However, as the team matures, leadership can arise also from within the team. Consequently, we do not view leadership as arising solely from the PI of team. A large body of work has shown that the practice of ***leadership*** has been associated with enhanced team performance [49,95]. Transformational leadership involves empowering team members to work towards a common, shared, goal [96]; a more relevant model is that of functional leadership, where satisfaction of the teams’ needs adapts to the stages of team development [49]. Leadership-specific KSAs that we identified include *conflict resolution, sense-making, goal-setting,* and *external networking*.

*Conflict Resolution.* Conflict arises when two or more members perceive the other’s actions as in opposition to its own. As with other team-emergent competencies, conflict is multidimensional and can manifest as task-based conflict and interpersonal conflict. Task-based conflict enhances effectiveness [97], whereas interpersonal conflict interferes with a team’s ability to collaborate and contributes to reduced satisfaction in team membership [98]. In clinical care teams, high rates of conflict are associated with significant medical errors and adverse patient outcomes [99]. Consequently, how a team deals with conflict significantly impacts its performance [100]. Methods of *conflict resolution* are based on the degree to which a leader’s practices emphasize cooperativeness or assertiveness [101]. Managing conflict cooperatively can lead to higher perceptions of fair treatment among individuals, which in turn leads to better team performance [102]. Perspective-seeking and promoting collaborative problem-solving may promote beneficial task-based conflict [103].

*Sense-making.* One important role team that leadership plays in responding to disruptive events is through the practice of *sense-making* [104-106]. In response to external disruptions, transformational leadership practices provide *sense-making* by shaping the meaning of work to team members [107]. *Sense-making* activities seek to turn an unexpected disruption into productive activity by providing insight into the event and developing a path forward. *Sense-making* frames a mental image of where the team is and where they are going in order to create an action plan in the face of uncertainty. By making meaning of these changes, teams are able to formulate a basis for action [108]. In this way, *sense-making* enables teams to respond to disruptive events through *learning/adaptation* [104-106].

*Goal-setting.* Leaders who set clear and challenging goals direct individual action and motivate individuals to achieve performance targets. At the team level, *goal-setting* helps teams form a common identity to enhance their commitment to team goals. Setting challenging goals enhances outcomes [109], energizing team members, and directing their attention [110–112]. Interestingly, identifying challenging goals is as important as leader behaviors that facilitate team creativity [109]. Consequently, teams with *goal-setting* outperform those without [68,69]. In addition to *goal-setting*, giving and providing feedback is important for team learning and performance. With feedback, shared visioning, and communication, members are more engaged, and the teams show evidence of increased cohesion [112,113].

*External networking.* Another role of leadership is providing interpersonal ties with external collaborators and networks. Leaders with central ties with external networks tend to have more productive teams. A meta-analysis of 37 studies suggests that teams with densely configured interpersonal ties attain their goals better [114]. Leadership has a significant positive impact on team learning behavior when time pressure is high [115], a circumstance that TTs often must navigate.

## II. Reinforcement Between Competency Domains

Our analysis confirms and clarifies our previous work on team-emergent competency “domains” by suggesting how practice in one domain reinforces practice in others. We note that ***affect*** *(trust) i*s foundational to all competency domains. Some of the strongest relationships identified in this review, consistent with prior work [4], are between those in the ***affect, communication,*** and ***leadership*** domains (Fig. 5). Leadership behaviors strongly influence the growth of a psychologically safe environment. Transformational leadership has a direct positive relationship with a safe team climate, which in turn promotes knowledge sharing and influences team members to trust one another by stimulating communication [21]. Risk-taking and cohesiveness within this environment encourages learning/adaptative behavior [12,17] and closely linked to team performance [116–118] and team member satisfaction [119]. In highly technical teams, shared leadership leverages a culture of knowledge sharing which helps the team to perform effective decision-making, problem-solving, and goal-setting by sharing their expertise and experiences [120]. These associations were replicated in a larger study of knowledge-based industry teams where transformational leadership was positively related to team member satisfaction and to objective team performance [121].

Additionally, competencies in ***communication*** influence ***affect*** and ***collaborative problem-solving***. In educational teams, ***communication*** promotes ***affect*** (trust) and ***learning/adaptive behaviors*** [122]. Leadership *sense-making* promotes *trust* and *conflict resolution* [123], where conflicting insights can be resolved and integrated into a common mental model [124].

## Skill-building Approaches

Having examined the specific 15 KSAs linked with team performance, we further examined the literature for approaches that would enhance behaviors of this competency (Table 1). The approaches below were evaluated on the strength of the evidence base on TTs using the Wisconsin Interventions in Team Science framework [125] and represent “empirically informed” strategies.

**Table 1.** Interventions for promoting Knowledge Skills and Attitudes. For each competency, interventions and proximal outcomes and behaviors are listed

Competency	Intervention	Outcome/Behavior
Trust	Leadership coaching-transformational leadership	Relational leadership-members connect with leadership
	Coaching-transformational leadership	Perspective-seeking in team interactions, conflict mediation/resolution
	Coaching	Psychological empowerment
Knowledge sharing	Coaching/training-sharing overarching vision of shared objective	Goal alignment; satisfaction in participating in team
	Coaching/training-leadership communication	Increase in intra-team advice network density
	Cross-training	Improved shared mental models
	Dedicated project management	Clarity of goals, outcomes and guidelines, data sharing
Transactive Memory	Conduct of interdependent projects	Convergence of *SMM*
	Method intuition	Understanding of skills, approaches, and biases of other disciplines on team
	Feedback	Team member efficacy, ability to adapt, resiliency
Team Members Roles Responsibilities	Collaboration planning	Team functioning, communication, conflict management, training, QI, transparency of resource allocation, team charter
	“Welcome to my Team” letters	Inclusion, conflict resolution, role definition
	Project Management Institute resources	Team meetings, goal clarity, change management
	Concept mapping/personnel inventory	Role definition, satisfaction in team
Shared Vision	Scientific retreats	Convergence of shared mental models, innovative experimental design
	Leadership serving as role model(s)	Satisfaction, member efficacy
	Team debriefs	Learning, improved team processes, QI
Learning/adaptation	Leadership knowledge sharing	Stakeholder engagement
	Team toolbox dialog	Shared understanding of perspectives and language
	Assessing and Teaching 21st Century Skills project assessment (ATC21S) of collaborative problem-solving	Assess ability to solve complex and interdisciplinary problems, leading to training opportunities
Sense-making	Steps of sense-making	Enhanced response to disruptive events

1. ***Affect.*** Facilitating TT ***affect*** can be highly influenced by leadership behaviors, behaviors that can be learned and coached [126]. A comprehensive meta-analysis of ∼5000 groups on the antecedents and outcomes of *psychological safety* found that positive leader interactions play a critical role in fostering an environment of inclusion and risk-taking [127]. Understanding styles, tendencies, recognizing strengths, and weaknesses is a component of inclusive leadership practice, promoting a feeling of team membership [128–130]. Transformational leaders who understand and listen to members promote trust, conflict resolution, and empowerment [123,131]. Diversity practices by the team leader have also been shown to promote ***affect***. In a study of >4500 health sector employees, ***Leadership*** diversity practices were found to enhance *trust* and *psychological safety* [132]. These interventions and proximal outcomes are shown in Table 1.
2. ***Communication.*** Simulation studies have found that cross-training enhances the development of *SMMs*, leading to enhanced performance[74]. Additionally, one impactful specific practice of transformational leadership, particularly during the formation of a TT, is that of “inspirational communication,” where a leader articulates an inspiring vision stressing the importance of collective action and of the mission to be accomplished (Table 1). A study of transformational leadership communication types comparing inspirational motivation or intellectual stimulation found that inspirational communication is linked to team performance and creativity [133]. Moreover, satisfaction of the teams goals and team performance was found to be associated with a multi-level trust – exhibited as both trust in the leader as well as trust in the team [134]. Finally, transformational leadership promotes within-team knowledge sharing and team innovative performance through an integration mechanism [135] and increasing knowledge sharing through the density of intra-team advice network density [136].
3. ***Management.*** A number of established approaches have been developed by professional societies to provide project management skills to TTs. The nontraditional role of the “project manager” can provide essential coordination for team activities; these skills have been empirically derived from CTSA-based TTs [80]. In addition, team charters describe an intervention used to develop team norms and processes and define various aspects of teamwork, including purpose and mission statements, operating guidelines, behavioral norms, and performance management practices [137,138]. Feedback enables adaptation and enhances long-term performance [68,77,113]. Empiric work has shown that shared leadership has a positive impact on team members’ innovative behavior [139] and ability to overcome barriers [140]. Feedback enables adaptation and enhances long-term performance [68,77,113]. Leadership behaviors can promote adaptation through leader briefings if team expertise can be developed and team knowledge enhanced [141]. We have recently developed a workshop approach to foster interdisciplinary collaboration known as “Collaboration Planning” [142]. Tested in 40 TTs, Collaboration Planning is a high-impact team-building activity that provides members with the skills to participate in collaborative TTs as well as promoting ***affect*** and a culture of reproducibility. Collaboration Planning is a ∼90-minute facilitated intervention in 10 areas, including Rationale for team approach and configuration, Collaboration Readiness, Technological Readiness, Team Functioning, Communication/coordination, Leadership/management/administration, Conflict prevention/management, Training, Quality Improvement and Resource Allocation [142]. Some of the unique aspects of the TT model that separate it from traditional product development teams are its dynamic membership and dependence on voluntary participation [3]. Although we noted that the TT has a stable core consisting of PI, trainee, and collaborating scientists (Fig. 1), additional scientists and community members associate with the team during its evolution. This association was dynamic, voluntary, and driven by the phase of translation [5]. New members can bring needed perspectives and technological talent, but also can be disruptive to many team activities. Approaches to vetting potential team members that have been found to be useful by NIH Ombudsman include interviews with prospective members focusing on values, performance, and behavior [143]. Collaborative agreement documents and “Welcome letters” are also approaches to reinforce expectations of working within a TT [143]. As new team members are brought into the project, focused onboarding activities, such as collaboration planning, and team-level training interventions may need to be repeated or made available in real-time.

1. ***Collaborative problem-solving.*** Promoting cognitive diversity is foundational to effective ***collaborative problem-solving***. During formation of the group, the toolbox dialog exercise is a workshop using a philosophical approach to promote interdisciplinary integration, promoting shared views, and language [144]. In established teams, Leadership practices that value and manage knowledge diversity are essential for engaging in learning that support the team goals [145,146] and effective team behaviors [147]. One approach, “perspective-taking,” is a collective cognitive process, through which team members strive to understand the world from other members’ viewpoints [148]. Perspective-taking increases proficiency, adaptivity, and proactivity when leaders adopt “both-and” approaches that behaviorally accept and integrate competing viewpoints [149]. Additionally, cognitive diversity is advanced by inclusive leadership behaviors that support sharing multiple viewpoints and mitigate hierarchal differences [128–130]. Inclusive leadership is positively linked with innovation in business teams [150,151] and potentially supports stakeholder engagement in TTs. An assessment tool for ***collaborative problem-solving*** has been applied in a 2015 international survey of 500,000 students in 52 countries, and training approaches have been proposed [11]. In this analysis, the specific team training strategies that can be related to team-focused training in knowledge sharing, critical thinking, and coordination have been shown to have some of the most important impact. More work will be required to adapt these training approaches to TTs and to evaluate their impact on performance or innovation.
2. ***Leadership.*** A number of leadership models have been developed in generic business and health care teams to promote *sense-making* and *goal-setting*. Taking from the business domain, the MIT Sloan School of Management has developed a stepped model for *sense-making* [152]. This approach employs 1. exploring the wider system; 2. creating a map of the current situation; and 3. acting to change the system to learn more about it. Consequently, teams are better able to adapt and respond to disruptive events (Table 1). Although sense-making approach has been developed for generic business teams, this approach will need to be adapted to the unique aspects of TTs.

## Discussion

The SciTS has benefitted substantially from the approach of drawing evidence from team research in education, social sciences, psychology, business, and medicine to identify characteristics that impact interdisciplinary team performance. In this study, we extend this successful approach for informing how to shape effective CTSA-type TTs. This consideration is important because we contend that TTs are a special case of interdisciplinary teams, with unique roles, competencies, and focus on product development goals. Here we extend previous work from the CTSA Team Science Affinity Group that identified five competency “domains” characteristic of successful TTs to team performance.

This review addresses an important gap by providing linkage between the competency “domain” and team performance, identifying KSAs within these domains. Our work proposes interventions that are linked to improvement in these TT-relevant KSAs. This work informs potential interventions in enhancing team performance in the CTSA environment. Many team science training interventions are focused on individual-focused training; however, we contend that the greatest impact on team performance will be on providing relevant training to teams on team-emergent skills that are strongly linked to performance. Training focused on teams is beginning to be developed, where training improves self-efficacy, leadership qualities in health care [153–155] and in the CTSA environment [7,126]. The extent to which these interventions can be applied to the leadership of TTs resulting in enhanced performance is just beginning to be evaluated [7].

Several themes emerge from our work, including a deeper understanding of the specific KSAs within a broad competency domain and specifics of their cross-reinforcing nature. One major finding of this study has been establishing the important relationship between a multi-level trusting environment (trust with the leadership and trust amongst team members) with knowledge sharing and leadership behaviors, resulting in enhanced team performance. Other interrelationships exist within the specific competencies as well. For example, within the knowledge sharing competency, a convergent *SMM* by itself is important, but insufficient for enhanced team performance, and needs to interact with a *transactive memory system* to produce enhanced team performance. Having shared clarity in the translational goal will not result in team performance unless members in the team understand “who” on the team can do “what.” Our review reveals a particularly powerful triad of the competency domains (Fig. 5) within which many exemplar combinations of KSAs might benefit a TT. As noted in the introduction, an inclusive environment (***affect***), openness to transdisciplinary knowledge sharing (***communication***), and situational leadership (***leadership***) exemplify a specific combination of KSAs within the triad of TS competency domains that reinforce and support one another.

Another important finding is that team-emergent competencies develop, evolve, and adapt over time. As team competencies first emerge, they could be viewed as rudimentary or “novice” in sophistication (Table 1). As the team matures its KSAs, these competencies are refined and made more advanced or “expert.” Some examples of this maturation of competencies could include the growth of individual trust into psychological safety, an evolution supported by the literature and noted above. Similarly, cohesion may have different manifestations between nascent and highly functional teams. For example, in nascent teams, only some members may exhibit shared emotional bonding, whereas in mature teams, this rapport may extend across all team members. Knowledge sharing is dependent on interdependent research activities conducted by the TT members during the conduct of their project. *Collaborative problem-solving* arises from *knowledge sharing*, communication, and cognitive diversity [94], leading to *collective intelligence* and *transdisciplinarity*. The understanding how team-based competencies change over team development will be a focus of subsequent work on how TTs form and mature.

An additional finding is the effect of leadership behaviors on virtually all of the competency domains associated with team performance. During the formation stages of the team, the source of leadership is primarily through the principal investigators. Although leadership is traditionally thought to be an individual role, observational studies have found that established teams have different leaders in different situations [156]. As a TT matures through the implementation phase, shared leadership behaviors may arise, where other team members provide needed expertise. This shared leadership enables TTs to enhance capacity and adapt to unforeseen challenges [49,157]. Moreover, leadership roles, responsibilities, and impact are determined by stages of team development [49,158,159]. A deeper analysis of how teams develop and leadership behaviors that promote the maturation and performance of TTs will need to be conducted.

Our focus in this analysis has been on the factors affecting TT performance, defined as output (knowledge, manuscripts, grants) and societal/clinical impact from translational intervention. This performance is one axis of an input-output-process model developed earlier to track the evolution of TTs [6], schematically illustrated in Fig. 2. This model is based on literature that team processes, many mapping to the 15 core competencies derived by our analysis, are closely linked with team performance. However, this model may be too simplistic. For example, we note that the practice of many competency domains affects other aspects of team function. For example, role clarity and setting challenging goals lead to enhanced satisfaction. Establishing a culture of psychological safety and collaborative problem-solving helps to establishing a culture of reproducibility, promoting reproducible research [160]. The practice of shared leadership promotes career development in trainees associated with a TT [5,80]. External network interactions conducted by knowledge brokers help to increase the impact of publications and dissemination of research findings [58]. The full scope and impact of effective KSAs on team outcomes, broadly defined, will require further empiric studies.

Our goal for this study is to capture the current state of knowledge related to team-emergent KSAs linked to performance. Our findings that a triad of an inclusive environment, openness to transdisciplinary knowledge sharing (communication), and situational leadership (leadership) reinforce and support one another can be subjected to empirical testing. Many questions need to be addressed – what are the trajectories of KSA development from “novice” to “expert” that develop as TT matures? Inherent in answering this question is a global understanding of TT development and maturation. What team-focused training modalities will result in application of these KSAs? Do these KSAs impact other domains? Empiric research on TTs across the CTSA consortium is sorely needed.

Teams operate within complex academic and clinical organizations, whose cultures, practices, and incentives significantly impact motivations for team science. This review did not focus on organizational climate, but more work will need to be done to understand TT-supportive academic environment. Some studies indicate that transformational leadership on project success via team-building is strengthened in organizations with higher levels of empowerment climate [161]. Additionally, positive Institutions that promote individual empathy enable transformational leadership to impact team performance [162]. More work on evaluating optimal practices on promoting TT effectiveness will need to be conducted.

In conclusion, our findings suggest that intact-team training focusing on the major KSAs of team performance, psychological safety and trust, communication/knowledge sharing, and leadership will be most impactful. Training approaches that account for the dynamic membership of TTs will need to be developed.

## Supplementary material

For supplementary material accompanying this paper visit https://doi.org/10.1017/cts.2023.17. click here to view supplementary material
