# Supplementary material for: Competencies supporting high-performance translational teams: A review of the SciTS evidence base
Source: J Clin Transl Sci. 2023 Mar 3;7(1):e62. doi: 10.1017/cts.2023.17 (PMC10052558; doi:10.1017/cts.2023.17)
Supplement: Supplementary file 1 [file ctssup.zip › S2059866123000171sup001.docx]

^1-146129-344^

1. Hoch, J.E., Pearce, C.L. & Welzel, L. Is the Most Effective Team Leadership Shared? The Impact of Shared Leadership, Age Diversity, and Coordination on Team Performance. *Journal of Personnel Psychology*. **9**, 105-116 (2010).

2. Künzle, B.*, et al*. Leadership in anaesthesia teams: The most effective leadership is shared. *Quality and Safety in Health Care*. **19**, (2010).

3. Dionne, S.D., Sayama, H., Hao, C. & Bush, B.J. The role of leadership in shared mental model convergence and team performance improvement: An agent-based computational model. *Leadership Quarterly*. **21**, 1035-1049 (2010).

4. Shondrick, S.J., Dinh, J.E. & Lord, R.G. Developments in implicit leadership theory and cognitive science: Applications to improving measurement and understanding alternatives to hierarchical leadership. *Leadership Quarterly*. **21**, 959-978 (2010).

5. Wang, X.H. & Howell, J.M. Exploring the Dual-Level Effects of Transformational Leadership on Followers. *J. Appl. Psychol.* **95**, 1134-1144 (2010).

6. Müller, R. & Turner, J.R. Attitudes and leadership competences for project success. *Baltic J. Manage.* **5**, 307-329 (2010).

7. Kathuria, R., Partovi, F.Y. & Greenhaus, J.H. Leadership practices, competitive priorities, and manufacturing group performance. *Int. J. Oper. Prod. Manage.* **30**, 1080-1105 (2010).

8. Künzle, B., Zala-Mezö, E., Kolbe, M., Wacker, J. & Grote, G. Substitutes for leadership in anaesthesia teams and their impact on leadership effectiveness. *Eur. J. Work Org. Psychol.* **19**, 505-531 (2010).

9. Sumner, M. & Slattery, D. The impact of leadership effectiveness and team processes on team performance in construction. *Int. J. Constr. Educ. Res.* **6**, 179-201 (2010).

10. Ragaz, N., Berk, A., Ford, D. & Morgan, M. Strategies for family health team leadership: lessons learned by successful teams. *Healthcare quarterly (Toronto, Ont.)*. **13**, 39-43 (2010).

11. Zander, L. & Butler, C.L. Leadership modes: Success strategies for multicultural teams. *Scandinavian Journal of Management*. **26**, 258-267 (2010).

12. Manning, T. & Robertson, B. Seniority and gender differences in 360-degree assessments of influencing, leadership and team behaviours. Part 2: Gender differences, conclusions and implications. *Industrial and Commercial Training*. **42**, 211-219 (2010).

13. Korek, S., Felfe, J. & Zaepernick-Rothe, U. Transformational leadership and commitment: A multilevel analysis of group-level influences and mediating processes. *Eur. J. Work Org. Psychol.* **19**, 364-387 (2010).

14. Tucker, S., Turner, N., Barling, J. & McEvoy, M. Transformational leadership and childrens' aggression in team settings: A short-term longitudinal study. *Leadership Quarterly*. **21**, 389-399 (2010).

15. Fitzgerald, S. & Schutte, N.S. Increasing transformational leadership through enhancing self-efficacy. *Journal of Management Development*. **29**, 495-505 (2010).

16. Taylor, H.A., Greene, B.R. & Filerman, G.L. A conceptual model for transformational clinical leadership within primary care group practice. *J. Ambul. Care Manage.* **33**, 97-107 (2010).

17. Fairfield, K.D. Growing up and growing out: Emerging adults learn management through service-learning. *J. Manage. Educ.* **34**, 113-141 (2010).

18. Zheng, W., Khoury, A.E. & Grobmeier, C. How do leadership and context matter in r&d team innovation?–a multiple case study. *Human Resource Development International*. **13**, 265-283 (2010).

19. Gupta, V.K., Huang, R. & Niranjan, S. A longitudinal examination of the relationship between Team Leadership and Performance. *J. Leadersh. Organ. Stud.* **17**, 335-350 (2010).

20. Carmeli, A. & Waldman, D.A. Leadership, behavioral context, and the performance of work groups in a knowledge-intensive setting. *Journal of Technology Transfer*. **35**, 384-400 (2010).

21. Morgeson, F.P., DeRue, D.S. & Karam, E.P. Leadership in teams: A functional approach to understanding leadership structures and processes. *Journal of Management*. **36**, 5-39 (2010).

22. Charbonnier-Voirin, A., El Akremi, A. & Vandenberghe, C. A Multilevel Model of Transformational Leadership and Adaptive Performance and the Moderating Role of Climate for Innovation. *Group & Organization Management*. **35**, 699-726 (2010).

23. Wu, J.B., Tsui, A.S. & Kinicki, A.J. Consequences of Differentiated Leadership in Groups. *Acad. Manage. J.* **53**, 90-106 (2010).

24. Cote, S., Lopes, P.N., Salovey, P. & Miners, C.T.H. Emotional intelligence and leadership emergence in small groups. *Leadership Quarterly*. **21**, 496-508 (2010).

25. Miles, J.R. & Kivlighan, D.M., Jr. Co-Leader Similarity and Group Climate in Group Interventions: Testing the Co-Leadership, Team Cognition-Team Diversity Model. *Group Dynamics-Theory Research and Practice*. **14**, 114-122 (2010).

26. Tuckman, B.W. Leadership Teams: Developing and Sustaining High Performance. *Management Decision*. **48**, 340-344 (2010).

27. Williams, H.M., Parker, S.K. & Turner, N. Proactively performing teams: The role of work design, transformational leadership, and team composition. *Journal of Occupational and Organizational Psychology*. **83**, 301-324 (2010).

28. Lee, P., Gillespie, N., Mann, L. & Wearing, A. Leadership and trust: Their effect on knowledge sharing and team performance. *Management Learning*. **41**, 473-491 (2010).

29. Hüttermann, H. & Boerner, S. Fostering innovation in functionally diverse teams: The two faces of transformational leadership. *Eur. J. Work Org. Psychol.* **20**, 833-854 (2011).

30. Kurt, T., Duyar, I. & Çalik, T. Are we legitimate yet?: A closer look at the casual relationship mechanisms among principal leadership, teacher self-efficacy and collective efficacy. *Journal of Management Development*. **31**, 71-86 (2011).

31. Vandewaerde, M., Voordeckers, W., Lambrechts, F. & Bammens, Y. Board Team Leadership Revisited: A Conceptual Model of Shared Leadership in the Boardroom. *Journal of Business Ethics*. **104**, 403-420 (2011).

32. Zhang, Z. & Peterson, S.J. Advice Networks in Teams: The Role of Transformational Leadership and Members' Core Self-Evaluations. *J. Appl. Psychol.* **96**, 1004-1017 (2011).

33. Rowold, J. Relationship between leadership behaviors and performance: The moderating role of a work team's level of age, gender, and cultural heterogeneity. *Leadersh. Organ. Dev. J.* **32**, 628-647 (2011).

34. Bradford, S.K. Leadership, collective efficacy and team performance: A new paradigm for the next generation. *Int. J. Web-Based Learn. Teach. Technol.* **6**, 35-45 (2011).

35. Bai, X. & Roberts, W. Taoism and its model of traits of successful leaders. *Journal of Management Development*. **30**, 724-739 (2011).

36. Schaubroeck, J., Lam, S.S.K. & Peng, A.C. Cognition-Based and Affect-Based Trust as Mediators of Leader Behavior Influences on Team Performance. *J. Appl. Psychol.* **96**, 863-871 (2011).

37. Hu, J. & Liden, R.C. Antecedents of Team Potency and Team Effectiveness: An Examination of Goal and Process Clarity and Servant Leadership. *J. Appl. Psychol.* **96**, 851-862 (2011).

38. Sullivan, R.S. Business schools and the innovation conundrum. *Journal of Management Development*. **30**, 492-498 (2011).

39. Tung, H.L. & Chang, Y.H. Effects of empowering leadership on performance in management team: Mediating effects of knowledge sharing and team cohesion. *Journal of Chinese Human Resources Management*. **2**, 43-60 (2011).

40. Boies, K., Lvina, E. & Martens, M.L. Shared leadership and team performance in a business strategy simulation. *Journal of Personnel Psychology*. **9**, 195-202 (2011).

41. Gifford, W., Davies, B., Tourangeau, A. & Lefebre, N. Developing team leadership to facilitate guideline utilization: Planning and evaluating a 3-month intervention strategy. *Journal of Nursing Management*. **19**, 121-132 (2011).

42. Zhang, X.-a., Cao, Q. & Tjosvold, D. Linking Transformational Leadership and Team Performance: A Conflict Management Approach. *Journal of Management Studies*. **48**, 1586-1611 (2011).

43. Davison, R.B., Hollenbeck, J.R., Barnes, C.M., Sleesman, D.J. & Ilgen, D.R. Coordinated Action in Multiteam Systems. *J. Appl. Psychol.* **97**, 808-824 (2012).

44. Yan, S.F., Gu, B. & Tang, Y.Q. The influences of transformational leadership on team innovation climate and team innovation performance. *Adv. Inf. Sci. Serv. Sci.* **4**, 132-140 (2012).

45. Walumbwa, F.O., Morrison, E.W. & Christensen, A.L. Ethical leadership and group in-role performance: The mediating roles of group conscientiousness and group voice. *Leadership Quarterly*. **23**, 953-964 (2012).

46. Wang, X.H.F. & Howell, J.M. A multilevel study of transformational leadership, identification, and follower outcomes. *Leadership Quarterly*. **23**, 775-790 (2012).

47. Ramthun, A.J. & Matkin, G.S. Multicultural Shared Leadership: A Conceptual Model of Shared Leadership in Culturally Diverse Teams. *J. Leadersh. Organ. Stud.* **19**, 303-314 (2012).

48. Colbert, A.E., Judge, T.A., Choi, D. & Wang, G. Assessing the trait theory of leadership using self and observer ratings of personality: The mediating role of contributions to group success. *Leadership Quarterly*. **23**, 670-685 (2012).

49. Wang, A.C., Hsieh, H.H., Tsai, C.Y. & Cheng, B.S. Does Value Congruence Lead to Voice? Cooperative Voice and Cooperative Silence under Team and Differentiated Transformational Leadership. *Management and Organization Review*. **8**, 341-370 (2012).

50. Hauschildt, K. & Konradt, U. Self-leadership and team members' work role performance. *J. Manage. Psychol.* **27**, 497-517 (2012).

51. Ishikawa, J. Transformational leadership and gatekeeping leadership: The roles of norm for maintaining consensus and shared leadership in team performance. *Asia Pacific Journal of Management*. **29**, 265-283 (2012).

52. Li, Y., Chun, H., Ashkanasy, N.M. & Ahlstrom, D. A multi-level study of emergent group leadership: Effects of emotional stability and group conflict. *Asia Pacific Journal of Management*. **29**, 351-366 (2012).

53. Nielsen, K. & Daniels, K. Does shared and differentiated transformational leadership predict followers' working conditions and well-being? *Leadership Quarterly*. **23**, 383-397 (2012).

54. Stoker, J.I., Grutterink, H. & Kolk, N.J. Do transformational CEOs always make the difference? The role of TMT feedback seeking behavior. *Leadership Quarterly*. **23**, 582-592 (2012).

55. Tumerman, M. & Hedberg Carlson, L.M. Increasing medical team cohesion and leadership behaviors using a 360-degree evaluation process. *Wis. Med. J.* **111**, 33-37 (2012).

56. Ishikawa, J. Leadership and performance in Japanese R&D teams. *Asia Pacific Business Review*. **18**, 241-258 (2012).

57. Gundersen, G., Hellesøy, B.T. & Raeder, S. Leading International Project Teams: The Effectiveness of Transformational Leadership in Dynamic Work Environments. *J. Leadersh. Organ. Stud.* **19**, 46-57 (2012).

58. Sanders, K. & Shipton, H. The relationship between transformational leadership and innovative behaviour in a healthcare context: A team learning versus a cohesion perspective. *European Journal of International Management*. **6**, 83-100 (2012).

59. Yang, L.R., Wu, K.S., Wang, F.K. & Chin, P.C. Relationships among project manager's leadership style, team interaction and project performance in the Taiwanese server industry. *Qual. Quant.* **46**, 207-219 (2012).

60. Chen, G., Farh, J.L., Campbell-Bush, E.M., Wu, Z. & Wu, X. Teams as innovative systems: Multilevel motivational antecedents of innovation in R&D teams. *J. Appl. Psychol.* **98**, 1018-1027 (2013).

61. Siassakos, D.*, et al*. What makes maternity teams effective and safe? Lessons from a series of research on teamwork, leadership and team training. *Acta Obstet. Gynecol. Scand.* **92**, 1239-1243 (2013).

62. Estrada, E. & Vargas-Estrada, E. How peer pressure shapes consensus, leadership, and innovations in social groups. *Sci. Rep.* **3**, (2013).

63. Owens, B.P., Johnson, M.D. & Mitchell, T.R. Expressed humility in organizations: Implications for performance, teams, and leadership. *Organization Science*. **24**, 1517-1538 (2013).

64. Muethel, M. & Hoegl, M. Shared leadership effectiveness in independent professional teams. *European Management Journal*. **31**, 423-432 (2013).

65. Eseryel, U.Y. & Eseryel, D. Action-embedded transformational leadership in self-managing global information systems development teams. *Journal of Strategic Information Systems*. **22**, 103-120 (2013).

66. Bonifas, R.P., Hedgpeth, J. & Kramer, C. Evidence of Empowerment in Resident Council Groups: An Examination of Two Leadership Models in Assisted Living. *Journal of Gerontological Social Work*. **56**, 281-298 (2013).

67. Cruz-Ortiz, V., Salanova, M. & Martínez, I.M. Transformational leadership and team performance: Linked by teamwork engagement. *Revista de Psicologia Social*. **28**, 183-196 (2013).

68. Daspit, J., Tillman, C.J., Boyd, N.G. & McKee, V. Cross-functional team effectiveness: An examination of internal team environment, shared leadership, and cohesion influences. *Team Performance Management*. **19**, 34-56 (2013).

69. Raes, E.*, et al*. Facilitating team learning through transformational leadership. *Instructional Science*. **41**, 287-305 (2013).

70. Braun, S., Peus, C., Weisweiler, S. & Frey, D. Transformational leadership, job satisfaction, and team performance: A multilevel mediation model of trust. *Leadership Quarterly*. **24**, 270-283 (2013).

71. Doyle, B.C. Civilian stabilization team leadership success: Perceptions for Iraq provincial reconstruction teams. *Stability*. **2**, (2013).

72. Taplin, S.H., Foster, M.K. & Shortell, S.M. Organizational leadership for building effective health care teams. *Ann. Fam. Med.* **11**, 279-281 (2013).

73. Hon, A.H.Y. & Chan, W.W.H. Team Creative Performance: The Roles of Empowering Leadership, Creative-Related Motivation, and Task Interdependence. *Cornell Hospitality Quarterly*. **54**, 199-210 (2013).

74. McIntyre, H.H. & Foti, R.J. The impact of shared leadership on teamwork mental models and performance in self-directed teams. *Group Processes Intergroup Relat.* **16**, 46-57 (2013).

75. Martínez-Córcoles, M., Gracia, F.J., Tomás, I., Peiró, J.M. & Schöbel, M. Empowering team leadership and safety performance in nuclear power plants: A multilevel approach. *Saf. Sci.* **51**, 293-301 (2013).

76. de Poel, F.M., Stoker, J.I. & Van der Zee, K.I. Leadership and Organizational Tenure Diversity as Determinants of Project Team Effectiveness. *Group Organ. Manage.* **39**, 532-560 (2014).

77. Ayoko, O.B. & Chua, E.L. The Importance of Transformational Leadership Behaviors in Team Mental Model Similarity, Team Efficacy, and Intra-Team Conflict. *Group Organ. Manage.* **39**, 504-531 (2014).

78. Somboonpakorn, A. & Kantabutra, S. Shared leadership and shared vision as predictors for team learning process, synergy and effectiveness in healthcare industry. *International Journal of Innovation and Learning*. **16**, 384-416 (2014).

79. Konradt, U. Toward a theory of dispersed leadership in teams: Model, findings, and directions for future research. *Leadership*. **10**, 289-307 (2014).

80. Lee, J., Lee, H. & Park, J.G. Exploring the Impact of Empowering Leadership on Knowledge Sharing, Absorptive Capacity and Team Performance in it Service. *Inf. Technol. People*. **27**, 366-386 (2014).

81. Chi, N.W. & Huang, J.C. Mechanisms Linking Transformational Leadership and Team Performance: The Mediating Roles of Team Goal Orientation and Group Affective Tone. *Group Organ. Manage.* **39**, 300-325 (2014).

82. Ahmed, N., Irshad, R. & Jamshaid, F.M. How emotional intelligence is related to team effectiveness? The mediating role of transformational leadership style. *World Appl. Sci. J.* **30**, 943-946 (2014).

83. Wang, D., Waldman, D.A. & Zhang, Z. A meta-analysis of shared leadership and team effectiveness. *J. Appl. Psychol.* **99**, 181-198 (2014).

84. Luciano, M.M., Mathieu, J.E. & Ruddy, T.M. Leading multiple teams: Average and relative external leadership influences on team empowerment and effectiveness. *J. Appl. Psychol.* **99**, 322-331 (2014).

85. Sun, W., Xu, A. & Shang, Y. Transformational leadership, team climate, and team performance within the NPD team: Evidence from China. *Asia Pacific Journal of Management*. **31**, 127-147 (2014).

86. Roberts, N.K.*, et al*. The impact of brief team communication, leadership and team behavior training on ad hoc team performance in trauma care settings. *Am. J. Surg.* **207**, 170-178 (2014).

87. Drescher, M.A., Audrey Korsgaard, M., Welpe, I.M., Picot, A. & Wigand, R.T. The dynamics of shared leadership: Building trust and enhancing performance. *J. Appl. Psychol.* **99**, 771-783 (2014).

88. Leicht, C., Randsley de Moura, G. & Crisp, R.J. Contesting gender stereotypes stimulates generalized fairness in the selection of leaders. *Leadership Quarterly*. **25**, 1025-1039 (2014).

89. Nicolaides, V.C.*, et al*. The shared leadership of teams: A meta-analysis of proximal, distal, and moderating relationships. *Leadership Quarterly*. **25**, 923-942 (2014).

90. Mitchell, R.*, et al*. Transformation through tension: The moderating impact of negative affect on transformational leadership in teams. *Human Relations*. **67**, 1095-1121 (2014).

91. Hayward, R.D. & Krause, N. Voluntary leadership roles in religious groups and rates of change in functional status during older adulthood. *J. Behav. Med.* **37**, 543-552 (2014).

92. Hoch, J.E. & Kozlowski, S.W.J. Leading virtual teams: Hierarchical leadership, structural supports, and shared team leadership. *J. Appl. Psychol.* **99**, 390-403 (2014).

93. Shin, Y. & Eom, C. Team proactivity as a linking mechanism between team creative efficacy, transformational leadership, and risk-taking norms and team creative performance. *Journal of Creative Behavior*. **48**, 89-114 (2014).

94. Jiang, W., Gu, Q. & Wang, G.G. To Guide or to Divide: The Dual-Side Effects of Transformational Leadership on Team Innovation. *J. Bus. Psychol.* **30**, 677-691 (2015).

95. Paunova, M. The emergence of individual and collective leadership in task groups: A matter of achievement and ascription. *Leadership Quarterly*. **26**, 935-957 (2015).

96. Cha, J., Kim, Y., Lee, J.Y. & Bachrach, D.G. Transformational Leadership and Inter-Team Collaboration: Exploring the Mediating Role of Teamwork Quality and Moderating Role of Team Size. *Group Organ. Manage.* **40**, 715-743 (2015).

97. Boies, K., Fiset, J. & Gill, H. Communication and trust are key: Unlocking the relationship between leadership and team performance and creativity. *Leadership Quarterly*. **26**, 1080-1094 (2015).

98. Lehmann-Willenbrock, N., Meinecke, A.L., Rowold, J. & Kauffeld, S. How transformational leadership works during team interactions: A behavioral process analysis. *Leadership Quarterly*. **26**, 1017-1033 (2015).

99. Zhang, X.A., Li, N., Ullrich, J. & van Dick, R. Getting Everyone on Board: The Effect of Differentiated Transformational Leadership by CEOs on Top Management Team Effectiveness and Leader-Rated Firm Performance. *Journal of Management*. **41**, 1898-1933 (2015).

100. Rao, A.S. & Kareem Abdul, W. Impact of transformational leadership on team performance: an empirical study in UAE. *Measuring Business Excellence*. **19**, 30-56 (2015).

101. Song, C., Park, K.R. & Kang, S.W. Servant leadership and team performance: The mediating role of knowledge-sharing climate. *Soc. Behav. Pers.* **43**, 1749-1760 (2015).

102. Rosenman, E.D., Ilgen, J.S., Shandro, J.R., Harper, A.L. & Fernandez, R. A Systematic Review of Tools Used to Assess Team Leadership in Health Care Action Teams. *Acad. Med.* **90**, 1408-1422 (2015).

103. De Hoogh, A.H.B., Greer, L.L. & Den Hartog, D.N. Diabolical dictators or capable commanders? An investigation of the differential effects of autocratic leadership on team performance. *Leadership Quarterly*. **26**, 687-701 (2015).

104. Schuller, K.A., Kash, B.A. & Gamm, L.D. Studer Group®'s evidence-based leadership initiatives: Comparing success and sustainability in two health systems. *Journal of Health Organization and Management*. **29**, 684-700 (2015).

105. Li, S.L., He, W., Yam, K.C. & Long, L.R. When and why empowering leadership increases followers’ taking charge: A multilevel examination in China. *Asia Pacific Journal of Management*. **32**, 645-670 (2015).

106. Waldman, D.A., Carter, M.Z. & Hom, P.W. A Multilevel Investigation of Leadership and Turnover Behavior. *Journal of Management*. **41**, 1724-1744 (2015).

107. Reuveni, Y. & Vashdi, D.R. Innovation in multidisciplinary teams: The moderating role of transformational leadership in the relationship between professional heterogeneity and shared mental models. *Eur. J. Work Org. Psychol.* **24**, 678-692 (2015).

108. Espinoza-Parra, S., Molero, F. & Fuster-Ruizdeapodaca, M.J. Transformational leadership and job satisfaction of police officers (carabineros) in Chile: the mediating effects of group identification and work engagement / Liderazgo transformacional y satisfacción laboral en carabineros de Chile: los efectos mediadores de la identificación con el grupo y el work engagement. *Revista de Psicologia Social*. **30**, 439-467 (2015).

109. Wu, C.H. & Wang, Z. How transformational leadership shapes team proactivity: The mediating role of positive affective tone and the moderating role of team task variety. *Group Dynamics*. **19**, 137-151 (2015).

110. To, M.L., Tse, H.H.M. & Ashkanasy, N.M. A multilevel model of transformational leadership, affect, and creative process behavior in work teams. *Leadership Quarterly*. **26**, 543-556 (2015).

111. Fernandez Castelao, E., Boos, M., Ringer, C., Eich, C. & Russo, S.G. Effect of CRM team leader training on team performance and leadership behavior in simulated cardiac arrest scenarios: a prospective, randomized, controlled study. *BMC Med. Educ.* **15**, (2015).

112. Odoardi, C., Montani, F., Boudrias, J.S. & Battistelli, A. Linking managerial practices and leadership style to innovative work behavior: The role of group and psychological processes. *Leadersh. Organ. Dev. J.* **36**, 545-569 (2015).

113. Forslund Frykedal, K. & Rosander, M. The role as moderator and mediator in parent education groups - a leadership and teaching approach model from a parent perspective. *Journal of Clinical Nursing*. **24**, 1966-1974 (2015).

114. Liff, R. & Wikström, E. The problem-avoiding multi professional team-On the need to overcome protective routines. *Scandinavian Journal of Management*. **31**, 266-278 (2015).

115. Mathieu, J.E., Kukenberger, M.R., D'Innocenzo, L. & Reilly, G. Modeling reciprocal team cohesion-performance relationships, as impacted by shared leadership and members' competence. *J. Appl. Psychol.* **100**, 713-734 (2015).

116. Sims, S., Hewitt, G. & Harris, R. Evidence of a shared purpose, critical reflection, innovation and leadership in interprofessional healthcare teams: A realist synthesis. *Journal of Interprofessional Care*. **29**, 209-215 (2015).

117. Liu, H.H. & Shieh, C.J. A study on the correlations among empowering leadership, organizational identification, and team performance in medical industry. *Studies on Ethno-Medicine*. **9**, 9-17 (2015).

118. Zacher, H. & Rosing, K. Ambidextrous leadership and team innovation. *Leadersh. Organ. Dev. J.* **36**, 54-68 (2015).

119. Brandt, T.M. & Edinger, P. Transformational leadership in teams–the effects of a team leader’s sex and personality. *Gender in Management*. **30**, 44-68 (2015).

120. Zhou, W., Vredenburgh, D. & Rogoff, E.G. Informational diversity and entrepreneurial team performance: moderating effect of shared leadership. *International Entrepreneurship and Management Journal*. **11**, 39-55 (2015).

121. Rossberger, R.J. & Krause, D.E. Participative and Team-Oriented Leadership Styles, Countries’ Education Level, and National Innovation: The Mediating Role of Economic Factors and National Cultural Practices. *Cross-Cultural Research*. **49**, 20-56 (2015).

122. Effelsberg, D. & Solga, M. Transformational Leaders’ In-Group versus Out-Group Orientation: Testing the Link Between Leaders’ Organizational Identification, their Willingness to Engage in Unethical Pro-Organizational Behavior, and Follower-Perceived Transformational Leadership. *Journal of Business Ethics*. **126**, 581-590 (2015).

123. Dizgah, M.R. & Keshavarz, A. Investigating the Relationship Between Emotional Intelligence, Transformational Leadership and Team Effectiveness in the Banks of Guilan Province-Iran. *Adv. Environ. Biol.* **9**, 992-1000 (2015).

124. Santos, J.P., Caetano, A. & Tavares, S.M. Is training leaders in functional leadership a useful tool for improving the performance of leadership functions and team effectiveness? *Leadership Quarterly*. **26**, 470-484 (2015).

125. Al Qubaisi, J.M.L.F., Abu Elanain, H.M., Badri, M.A. & Ajmal, M.M. Leadership, culture and team communication: Analysis of project success causality - A UAE case. *Int. J. Appl. Manage. Sci.* **7**, 223-243 (2015).

126. Chen, Z., Zhu, J. & Zhou, M. How does a servant leader fuel the service fire? A multilevel model of servant leadership, individual self identity, group competition climate, and customer service performance. *J. Appl. Psychol.* **100**, 511-521 (2015).

127. Balamohan, P., Tech, M. & Gomathi, S. Emotional intelligence – Its importance and relationship with individual peformance,team-effectiveness, leadership and marketing effectiveness. *Mediterranean J. Soc. Sci.* **6**, 120-128 (2015).

128. Marchiondo, L.A., Myers, C.G. & Kopelman, S. The relational nature of leadership identity construction: How and when it influences perceived leadership and decision-making. *Leadership Quarterly*. **26**, 892-908 (2015).

129. García-Guiu, C., Moya, M., Molero, F. & Moriano, J.A. Transformational leadership and group potency in small military units: The mediating role of group identification and cohesion. *Rev. Psicol. Trab. Orga.* **32**, 145-152 (2016).

130. Chiu, C.Y.C., Owens, B.P. & Tesluk, P.E. Initiating and utilizing shared leadership in teams: The role of leader humility, team proactive personality, and team performance capability. *J. Appl. Psychol.* **101**, 1705-1720 (2016).

131. D’Innocenzo, L., Mathieu, J.E. & Kukenberger, M.R. A Meta-Analysis of Different Forms of Shared Leadership–Team Performance Relations. *Journal of Management*. **42**, 1964-1991 (2016).

132. Meyer, B.*, et al*. What good leaders actually do: micro-level leadership behaviour, leader evaluations, and team decision quality. *Eur. J. Work Org. Psychol.* **25**, 773-789 (2016).

133. Charlier, S.D., Stewart, G.L., Greco, L.M. & Reeves, C.J. Emergent leadership in virtual teams: A multilevel investigation of individual communication and team dispersion antecedents. *Leadership Quarterly*. **27**, 745-764 (2016).

134. Bai, Y., Lin, L. & Li, P.P. How to enable employee creativity in a team context: A cross-level mediating process of transformational leadership. *Journal of Business Research*. **69**, 3240-3250 (2016).

135. Wang, X.H., Kim, T.Y. & Lee, D.R. Cognitive diversity and team creativity: Effects of team intrinsic motivation and transformational leadership. *Journal of Business Research*. **69**, 3231-3239 (2016).

136. Barnett, R.C. & Weidenfeller, N.K. Shared Leadership and Team Performance. *Advances in Developing Human Resources*. **18**, 334-351 (2016).

137. Banks, G.C., McCauley, K.D., Gardner, W.L. & Guler, C.E. A meta-analytic review of authentic and transformational leadership: A test for redundancy. *Leadership Quarterly*. **27**, 634-652 (2016).

138. Santos, C.M., Passos, A.M., Uitdewilligen, S. & Nübold, A. Shared temporal cognitions as substitute for temporal leadership: An analysis of their effects on temporal conflict and team performance. *Leadership Quarterly*. **27**, 574-587 (2016).

139. Aga, D.A., Noorderhaven, N. & Vallejo, B. Transformational leadership and project success: The mediating role of team-building. *International Journal of Project Management*. **34**, 806-818 (2016).

140. Vanderboom, C.E.*, et al*. Leadership strategies, an interdisciplinary team, and ongoing nurse feedback: Ingredients for a successful BCMA project. *Nurs. Econ.* **34**, 117-125 (2016).

141. Lauck, S.B., McGladrey, J., Lawlor, C. & Webb, J.G. Nursing leadership of the transcatheter aortic valve implantation Heart Team: Supporting innovation, excellence, and sustainability. *Healthc. Manage. Forum*. **29**, 126-130 (2016).

142. Ekpe, I. & Mat, N. Can academic performance enhance group membership and leadership among student entrepreneurs in Malaysia? *Asian Soc. Sci.* **12**, 87-92 (2016).

143. Quinteiro, P.M., Passos, A. & Curral, L. Thought self-leadership and effectiveness in self-management teams. *Leadership*. **12**, 110-126 (2016).

144. Li, V., Mitchell, R. & Boyle, B. The Divergent Effects of Transformational Leadership on Individual and Team Innovation. *Group Organ. Manage.* **41**, 66-97 (2016).

145. Losch, S., Traut-Mattausch, E., Mühlberger, M.D. & Jonas, E. Comparing the effectiveness of individual coaching, self-coaching, and group training: How leadership makes the difference. *Front. Psychol.* **7**, (2016).

146. Sousa, M. & Van Dierendonck, D. Introducing a short measure of shared servant leadership impacting team performance through team behavioral integration. *Front. Psychol.* **6**, (2016).

147. Rosenman, E.D., Branzetti, J.B. & Fernandez, R. Assessing Team Leadership in Emergency Medicine: The Milestones and Beyond. *J. Grad. Med. Educ.* **8**, 332-340 (2016).

148. Diversity as a contributor to leadership effectiveness: Leadership styles to optimize employee skills and group outcomes. *Strateg. Dir.* **32**, 26-28 (2016).

149. Haselberger, D. A literature-based framework of performance-related leadership interactions in ICT project teams. *Information and Software Technology*. **70**, 1-17 (2016).

150. Owens, B.P. & Hekman, D.R. How Does Leader Humility Influence Team Performance? Exploring the Mechanisms of Contagion and Collective Promotion Focus. *Acad. Manage. J.* **59**, 1088-1111 (2016).

151. Friedrich, T.L., Griffith, J.A. & Mumford, M.D. Collective leadership behaviors: Evaluating the leader, team network, and problem situation characteristics that influence their use. *Leadership Quarterly*. **27**, 312-333 (2016).

152. Drescher, G. & Garbers, Y. Shared leadership leadership and commonality: A policy-capturing study. *Leadership Quarterly*. **27**, 200-217 (2016).

153. Parry, K., Cohen, M. & Bhattacharya, S. Rise of the Machines: A Critical Consideration of Automated Leadership Decision Making in Organizations. *Group & Organization Management*. **41**, 571-594 (2016).

154. Willems, J. Building Shared Mental Models of Organizational Effectiveness in Leadership Teams Through Team Member Exchange Quality. *Nonprofit and Voluntary Sector Quarterly*. **45**, 568-592 (2016).

155. Chun, J.U., Cho, K. & Sosik, J.J. A multilevel study of group-focused and individual-focused transformational leadership, social exchange relationships, and performance in teams. *Journal of Organizational Behavior*. **37**, 374-396 (2016).

156. Mukherjee, S. Leadership network and team performance in interactive contests. *Social Networks*. **47**, 85-92 (2016).

157. Zhou, J., Liu, S., Zhang, X. & Chen, M. Differential leadership, team conflict and new product development performance An empirical study from R&D team in China. *Chinese Management Studies*. **10**, 544-558 (2016).

158. Benoliel, P. & Somech, A. Functional heterogeneity and senior management team effectiveness The mediating role of school leadership. *Journal of Educational Administration*. **54**, 492-512 (2016).

159. Hill, N.S. & Bartol, K.M. Empowering Leadership and Effective Collaboration in Geographically Dispersed Teams. *Pers. Psychol.* **69**, 159-198 (2016).

160. De Jong, B.A., Dirks, K.T. & Gillespie, N. Trust and team performance: A meta-analysis of main effects, moderators, and covariates. *J. Appl. Psychol.* **101**, 1134-1150 (2016).

161. Guenter, H., Gardner, W.L., Davis McCauley, K., Randolph-Seng, B. & Prabhu, V.P. Shared Authentic Leadership in Research Teams: Testing a Multiple Mediation Model. *Small Group Research*. **48**, 719-765 (2017).

162. Mayr, M.L. Transformational Leadership and Volunteer Firefighter Engagement: The Mediating Role of Group Identification and Perceived Social Impact. *Nonprofit Manag. Leadersh.* **28**, 259-270 (2017).

163. Karriker, J.H., Madden, L.T. & Katell, L.A. Team Composition, Distributed Leadership, and Performance: It’s Good to Share. *J. Leadersh. Organ. Stud.* **24**, 507-518 (2017).

164. Travis Maynard, M., Resick, C.J., Cunningham, Q.W. & DiRenzo, M.S. Ch-Ch-Ch-changes: How Action Phase Functional Leadership, Team Human Capital, and Interim vs. Permanent Leader Status Impact Post-transition Team Performance. *J. Bus. Psychol.* **32**, 575-593 (2017).

165. Lopez-Zafra, E., Pulido-Martos, M., Berrios-Martos, P. & Augusto-Landa, J.M. Does transformational leadership predict group emotional intelligence in work settings? / ¿Es el liderazgo transformacional un predictor de la inteligencia emocional grupal en los contextos laborales? *Revista de Psicologia Social*. **32**, 513-538 (2017).

166. Leuteritz, J.P., Navarro, J. & Berger, R. How knowledge worker teams deal effectively with task uncertainty: The impact of transformational leadership and group development. *Front. Psychol.* **8**, (2017).

167. Zhou, W., Zhang, Y. & Shen, Y. How shared leadership and team personality composition interact to improve entrepreneurial team performance: Evidence from China. *Journal of Small Business and Enterprise Development*. **24**, 426-445 (2017).

168. Bouwmans, M., Runhaar, P., Wesselink, R. & Mulder, M. Fostering teachers' team learning: An interplay between transformational leadership and participative decision-making? *Teaching and Teacher Education*. **65**, 71-80 (2017).

169. Turner, J.R. & Baker, R. Team Emergence Leadership Development and Evaluation: A Theoretical Model Using Complexity Theory. *J. Inf. Knowl. Manage.* **16**, (2017).

170. Diebig, M., Bormann, K.C. & Rowold, J. Day-level transformational leadership and followers’ daily level of stress: a moderated mediation model of team cooperation, role conflict, and type of communication. *Eur. J. Work Org. Psychol.* **26**, 234-249 (2017).

171. Ceri-Booms, M., Curşeu, P.L. & Oerlemans, L.A.G. Task and person-focused leadership behaviors and team performance: A meta-analysis. *Human Resource Management Review*. **27**, 178-192 (2017).

172. Dong, Y., Bartol, K.M., Zhang, Z.X. & Li, C. Enhancing employee creativity via individual skill development and team knowledge sharing: Influences of dual-focused transformational leadership. *Journal of Organizational Behavior*. **38**, 439-458 (2017).

173. Qi, L. & Liu, B. Effects of Inclusive Leadership on Employee Voice Behavior and Team Performance: The Mediating Role of Caring Ethical Climate. *Front. Commun.* **2**, (2017).

174. Seelhofer, D. & Valeri, G. The interplay between leadership and team performance: An empirical investigation in a major Swiss HR consulting firm. *Central European Business Review*. **6**, 5-25 (2017).

175. Huvane, J.*, et al*. Fundamentals and catalytic innovation: The statistical and data management center of the antibacterial resistance leadership group. *Clin. Infect. Dis.* **64**, S18-S23 (2017).

176. Cross, H.R., Harris, A., Arias, R.M., Chambers, H.F. & Fowler, V.G., Jr. Transforming concepts into clinical trials and creating a multisite network: The Leadership and Operations Center of the Antibacterial Resistance Leadership Group. *Clin. Infect. Dis.* **64**, S8-S12 (2017).

177. Manca, C.*, et al*. Leading antibacterial laboratory research by integrating conventional and innovative approaches: The Laboratory Center of the Antibacterial Resistance Leadership Group. *Clin. Infect. Dis.* **64**, S13-S17 (2017).

178. Aw, V.K.J. & Ayoko, O.B. The impact of followers’ conflict behaviors on teams’ transformational leadership, team member exchange and engagement. *International Journal of Conflict Management*. **28**, 509-532 (2017).

179. Donnelly, L.F. Aspirational characteristics for effective leadership of improvement teams. *Pediatr. Radiol.* **47**, 17-21 (2017).

180. Pratoom, K. Differential Relationship of Person- and Task-Focused Leadership to Team Effectiveness: A Meta-Analysis of Moderators. *Human Resource Development Review*. **17**, 393-439 (2018).

181. Rahmani, M., Roels, G. & Karmarkar, U.S. Team leadership and performance: Combining the roles of direction and contribution. *Management Science*. **64**, 5234-5249 (2018).

182. Seong, J.Y. & Hong, D.S. Age diversity, group organisational citizenship behaviour, and group performance: Exploring the moderating role of charismatic leadership and participation in decision-making. *Human Resource Management Journal*. **28**, 621-640 (2018).

183. Rebelo, T., Dimas, I.D., Lourenço, P.R. & Palácio, Â. Generating team PsyCap through transformational leadership: A route to team learning and performance. *Team Performance Management*. **24**, 363-379 (2018).

184. Shokory, S.M. & Suradi, N.R.M. Transformational leadership and its impact on extra-role performance of project team members: The mediating role of work engagement. *Acad. Strateg. Manage. J*. **17**, (2018).

185. Schaubroeck, J.M.*, et al*. Changing experiences of work dirtiness, occupational disidentification, and employee withdrawal. *J. Appl. Psychol.* **103**, 1086-1100 (2018).

186. Han, J.H., Liao, H., Taylor, M.S. & Kim, S. Effects of high-performance work systems on transformational leadership and team performance: Investigating the moderating roles of organizational orientations. *Hum. Resour. Manage.* **57**, 1065-1082 (2018).

187. Li, G., Liu, H. & Luo, Y. Directive versus participative leadership: Dispositional antecedents and team consequences. *Journal of Occupational and Organizational Psychology*. **91**, 645-664 (2018).

188. Carter, M.Z., Mossholder, K.W. & Harris, J.N. Congruence effects of contingent reward leadership intended and experienced on team effectiveness: The mediating role of distributive justice climate. *Journal of Occupational and Organizational Psychology*. **91**, 465-485 (2018).

189. Dimas, I.D., Rebelo, T., Lourenço, P.R. & Pessoa, C.I.P. Bouncing Back from Setbacks: On the Mediating Role of Team Resilience in the Relationship Between Transformational Leadership and Team Effectiveness. *J. Psychol. Interdiscip. Appl.* **152**, 358-372 (2018).

190. Liu, H. & Li, G. Linking Transformational Leadership and Knowledge Sharing: The Mediating Roles of Perceived Team Goal Commitment and Perceived Team Identification. *Front. Psychol.* **9**, (2018).

191. Tu, M.H., Bono, J.E., Shum, C. & LaMontagne, L. Breaking the cycle: The effects of role model performance and ideal leadership self-concepts on abusive supervision spillover. *J. Appl. Psychol.* **103**, 689-702 (2018).

192. Knipfer, K., Schreiner, E., Schmid, E. & Peus, C. The Performance of Pre-Founding Entrepreneurial Teams: The Importance of Learning and Leadership. *Appl. Psychol.* **67**, 401-427 (2018).

193. Han, S.J., Lee, Y., Beyerlein, M. & Kolb, J. Shared leadership in teams: The role of coordination, goal commitment, and knowledge sharing on perceived team performance. *Team Performance Management*. **24**, 150-168 (2018).

194. Choi, E.H., Kim, E.K. & Kim, P.B. Effects of the Educational Leadership of Nursing Unit Managers on Team Effectiveness: Mediating Effects of Organizational Communication. *Asian Nurs. Res. (Korean Soc. Nurs. Sci.)*. **12**, 99-105 (2018).

195. Jiang, Y. & Chen, C.C. Integrating Knowledge Activities for Team Innovation: Effects of Transformational Leadership. *Journal of Management*. **44**, 1819-1847 (2018).

196. Lai, C.Y., Hsu, J.S.C. & Li, Y. Leadership, regulatory focus and information systems development project team performance. *International Journal of Project Management*. **36**, 566-582 (2018).

197. Chen, Q. & Liu, Z. How does TMT transactive memory system drive innovation ambidexterity?: Shared leadership as mediator and team goal orientations as moderators. *Chinese Management Studies*. **12**, 125-147 (2018).

198. Ye, X., Feng, J., Ma, L. & Huang, X. Impact of team leadership habitual domains on ambidextrous innovation. *Soc. Behav. Pers.* **46**, 1955-1966 (2018).

199. Paolucci, N., Dimas, I.D., Zappalà, S., Lourenço, P.R. & Rebelo, T. Transformational leadership and team effectiveness: The mediating role of affective team commitment. *Rev. Psicol. Trab. Orga.* **34**, 135-144 (2018).

200. Varela Medina, N.D. & González Macías, C.J. Perception of transformational leadership style and its effectiveness on virtual work-teams (VWT). A literature review in the organizational context. *Espacios*. **39**, (2018).

201. Maduka, N.S., Edwards, H., Greenwood, D., Osborne, A. & Babatunde, S.O. Analysis of competencies for effective virtual team leadership in building successful organisations. *Benchmarking*. **25**, 696-712 (2018).

202. Barling, J., Akers, A. & Beiko, D. The impact of positive and negative intraoperative surgeons’ leadership behaviors on surgical team performance. *Am. J. Surg.* **215**, 14-18 (2018).

203. Sperber, S. & Linder, C. The impact of top management teams on firm innovativeness: a configurational analysis of demographic characteristics, leadership style and team power distribution. *Review of Managerial Science*. **12**, 285-316 (2018).

204. Zhu, J., Yao, J. & Zhang, L. Linking empowering leadership to innovative behavior in professional learning communities: the role of psychological empowerment and team psychological safety. *Asia Pacific Education Review*. **20**, 657-671 (2019).

205. Zhang, L. & Guo, H. Enabling knowledge diversity to benefit cross-functional project teams: Joint roles of knowledge leadership and transactive memory system. *Information and Management*. **56**, (2019).

206. Nassif, A.G. Heterogeneity and centrality of “dark personality” within teams, shared leadership, and team performance: A conceptual moderated-mediation model. *Human Resource Management Review*. **29**, (2019).

207. Zhu, F., Wang, L., Yu, M., Müller, R. & Sun, X. Transformational leadership and project team members’ silence: the mediating role of feeling trusted. *International Journal of Managing Projects in Business*. **12**, 845-868 (2019).

208. Balwant, P. Stay close! The role of leader distance in the relationship between transformational leadership, work engagement, and performance in undergraduate project teams. *Journal of Education for Business*. **94**, 369-380 (2019).

209. Huang, S., Chen, J., Mei, L. & Mo, W. The effect of heterogeneity and leadership on innovation performance: Evidence from university research teams in China. *Sustainability (Switzerland)*. **11**, (2019).

210. Storm, C.P. & Scheepers, C.B. The Impact of Perceived Work Complexity and Shared Leadership on Team Performance of IT Employees of South African Firms. *Information Systems Management*. **36**, 195-211 (2019).

211. Eisenberg, J., Post, C. & DiTomaso, N. Team Dispersion and Performance: The Role of Team Communication and Transformational Leadership. *Small Group Research*. **50**, 348-380 (2019).

212. Scott-Young, C.M., Georgy, M. & Grisinger, A. Shared leadership in project teams: An integrative multi-level conceptual model and research agenda. *International Journal of Project Management*. **37**, 565-581 (2019).

213. Mitchell, R. & Boyle, B. Inspirational leadership, positive mood, and team innovation: A moderated mediation investigation into the pivotal role of professional salience. *Hum. Resour. Manage.* **58**, 269-283 (2019).

214. Nandana Prabhu, K.P., Rodrigues, L.L.R. & Pai, Y. Transformational leadership and workplace spirituality: A structural model of team effectiveness. *Prabandhan Ind. J. Manag.* **12**, 7-22 (2019).

215. Tschan, F., Semmer, N.K., Vetterli, M., Hunziker, P.R. & Marsch, S.C. Predicting team-performance and leadership in emergency situations by observing standardised operational procedures: A prospective single-blind simulator-based trial. *BMJ Simul. Technol. Enhanc. Learning*. **5**, 102-107 (2019).

216. Lin, C.P., Wang, C.C., Chen, S.C. & Chen, J.Y. Modeling leadership and team performance: The mediation of collective efficacy and the moderation of team justice. *Personnel Review*. **48**, 471-491 (2019).

217. Heinemann, A.W.*, et al*. Effects of an Implementation Intervention to Promote Use of Patient-Reported Outcome Measures on Clinicians' Perceptions of Evidence-Based Practice, Implementation Leadership, and Team Functioning. *J. Contin. Educ. Health Prof.* **39**, 103-111 (2019).

218. Lee, S.M. & Farh, C.I.C. Dynamic leadership emergence: Differential impact of members' and peers' contributions in the idea generation and idea enactment phases of innovation project teams. *J. Appl. Psychol.* **104**, 411-432 (2019).

219. Lorinkova, N.M. & Perry, S.J. The importance of group-focused transformational leadership and felt obligation for helping and group performance. *Journal of Organizational Behavior*. **40**, 231-247 (2019).

220. Wong, S.I. & Berntzen, M.N. Transformational leadership and leader–member exchange in distributed teams: The roles of electronic dependence and team task interdependence. *Comput. Human Behav.* **92**, 381-392 (2019).

221. Bachrach, D.G. & Mullins, R. A dual-process contingency model of leadership, transactive memory systems and team performance. *Journal of Business Research*. **96**, 297-308 (2019).

222. Zhou, L., Wang, M. & Vancouver, J.B. A Formal Model of Leadership Goal Striving: Development of Core Process Mechanisms and Extensions to Action Team Context. *J. Appl. Psychol.* **104**, 388-410 (2019).

223. Yang, F., Huang, X. & Wu, L. Experiencing meaningfulness climate in teams: How spiritual leadership enhances team effectiveness when facing uncertain tasks. *Hum. Resour. Manage.* **58**, 155-168 (2019).

224. Bachrach, D.G.*, et al*. Transactive memory systems in context: A meta-analytic examination of contextual factors in transactive memory systems development and team performance. *J. Appl. Psychol.* **104**, 464-493 (2019).

225. Lee, E.K., Avgar, A.C., Park, W.W. & Choi, D. The dual effects of task conflict on team creativity: Focusing on the role of team-focused transformational leadership. *International Journal of Conflict Management*. **30**, 132-154 (2019).

226. Eva, N., Newman, A., Miao, Q., Cooper, B. & Herbert, K. Chief executive officer participative leadership and the performance of new venture teams. *Int. Small. Bus. J.* **37**, 69-88 (2019).

227. Bruccoleri, M., Riccobono, F. & Größler, A. Shared Leadership Regulates Operational Team Performance in the Presence of Extreme Decisional Consensus/Conflict: Evidences from Business Process Reengineering. *Decision Sciences*. **50**, 46-83 (2019).

228. Nordbäck, E.S. & Espinosa, J.A. Effective Coordination of Shared Leadership in Global Virtual Teams. *Journal of Management Information Systems*. **36**, 321-350 (2019).

229. Kim, B.J., Park, S. & Kim, T.H. The effect of transformational leadership on team creativity: sequential mediating effect of employee’s psychological safety and creativity. *Asian Journal of Technology Innovation*. **27**, 90-107 (2019).

230. Novoselich, B.J. & Knight, D.B. Relating shared leadership to capstone team effectiveness. *International Journal of Engineering Education*. **35**, 1888-1906 (2019).

231. Watthanabut, B. Knowledge exchange and transformational leadership style for team improvement. *Utopia Prax. Latinoamericana*. **24**, 207-214 (2019).

232. Garcia, F.A.Z. & Russo, R.F.S.M. Leadership and performance of the software development team: Influence of the type of project management. *Rev. Bras. Gestao Negocios*. **21**, 970-1005 (2019).

233. Wegge, J., Jungbauer, K.L. & Shemla, M. When inspiration does not fit the bill: Charismatic leadership reduces performance in a team crisis for followers high in self-direction. *J. Manage. Organ.* (2019).

234. Othman, F.S., Saad, M.S.M. & Robani, A. The impact of leadership styles on r&d team performance and the mediating role of knowledge sharing. *Humanit. Soc. Sci. Rev.* **7**, 307-314 (2019).

235. Vojvodic, M. & Hitz, C. Governance team leadership and business user participation - Organizational practices for innovative customer engagement in data compliance project. *Central European Business Review*. **8**, 15-45 (2019).

236. Ul Hassan, M., Iqbal, Z., Shafique, U. & Bukhari, N. Transformational leadership and team performance: An empirical study of the hotel industry of Pakistan. *Int. J. Bus. Excellence*. **18**, 151-173 (2019).

237. Lee, M.H. & Ko, K.M. The influence of supervisors’ leadership skills and team cohesion on team performance in environmental service industry. *Ekoloji*. **28**, 1013-1017 (2019).

238. Leppin, A.L.*, et al*. Situating dissemination and implementation sciences within and across the translational research spectrum. *J Clin Transl Sci*. **4**, 152-158 (2019).

239. Klaic, A., Burtscher, M.J. & Jonas, K. Fostering team innovation and learning by means of team-centric transformational leadership: The role of teamwork quality. *Journal of Occupational and Organizational Psychology*. **93**, 942-966 (2020).

240. Bruning, P.F., Turner, N. & Lin, H.C. Transformational leadership, group affective tone, and group member social inferences: A leadership complementarity perspective. *Can. J. Adm. Sci.* **37**, 383-395 (2020).

241. Chen, Z., Chen, Z., Yu, Y. & Huang, S. How Shared Leadership in Entrepreneurial Teams Influences New Venture Performance: A Moderated Mediation Model. *J. Leadersh. Organ. Stud.* **27**, 406-418 (2020).

242. Anselmann, V. & Mulder, R.H. Transformational leadership, knowledge sharing and reflection, and work teams’ performance: A structural equation modelling analysis. *Journal of Nursing Management*. **28**, 1627-1634 (2020).

243. Homan, A.C., Gündemir, S., Buengeler, C. & van Kleef, G.A. Integrative conceptual review: Leading diversity: Towards a theory of functional leadership in diverse teams. *J. Appl. Psychol.* **105**, 1101-1128 (2020).

244. Kelemen, T.K., Matthews, S.H., Zhang, X.A., Bradley, B.H. & Liu, H. When does gender diversity enhance team performance? The dual need for visionary leadership and team tenure. *J. Appl. Soc. Psychol.* **50**, 501-511 (2020).

245. Han, J.H., Liao, H., Kim, S. & Han, J. Narcissism and Empowerment: How Narcissism Influences the Trickle-Down Effects of Organizational Empowerment Climate on Performance. *Journal of Management Studies*. **57**, 1217-1245 (2020).

246. Kindarto, A., Zhu, Y.Q. & Gardner, D.G. Full Range Leadership Styles and Government IT Team Performance: The Critical Roles of Follower and Team Competence. *Public Perform. Manage. Rev.* **43**, 889-917 (2020).

247. Tang, G., Chen, Y., van Knippenberg, D. & Yu, B. Antecedents and consequences of empowering leadership: Leader power distance, leader perception of team capability, and team innovation. *Journal of Organizational Behavior*. **41**, 551-566 (2020).

248. Mysirlaki, S. & Paraskeva, F. Emotional intelligence and transformational leadership in virtual teams: lessons from MMOGs. *Leadersh. Organ. Dev. J.* **41**, 551-566 (2020).

249. Cheung, C.M. & Zhang, R.P. How Organizational Support Can Cultivate a Multilevel Safety Climate in the Construction Industry. *J. Manage. Eng.* **36**, (2020).

250. Mahdikhani, M. & Yazdani, B. Transformational leadership and service quality in e-commerce businesses: The role of trust and team performance. *International Journal of Law and Management*. **62**, 23-46 (2020).

251. Cavazotte, F., Moreno, V. & Lasmar, L.C.C. Enabling customer satisfaction in call center teams: the role of transformational leadership in the service-profit chain. *Service Industries Journal*. **40**, 380-393 (2020).

252. Pallesen, K.S., Rogers, L., Anjara, S., De Brún, A. & McAuliffe, E. A qualitative evaluation of participants' experiences of using co-design to develop a collective leadership educational intervention for health-care teams. *Health Expectations*. **23**, 358-367 (2020).

253. Cook, A.S., Zill, A. & Meyer, B. Observing leadership as behavior in teams and herds – An ethological approach to shared leadership research. *Leadership Quarterly*. **31**, (2020).

254. Dwidienawati, D., Arief, M., Gandasari, D. & Pradipto, Y.D. Ambidextrous leadership influent toce on team creativity, team innovation and team performance. *Int. J. Adv. Sci. Technol.* **29**, 6268-6279 (2020).

255. Siangchokyoo, N., Klinger, R.L. & Campion, E.D. Follower transformation as the linchpin of transformational leadership theory: A systematic review and future research agenda. *Leadership Quarterly*. **31**, (2020).

256. Willgerodt, M.A.*, et al*. Impact of leadership development workshops in facilitating team-based practice transformation. *Journal of Interprofessional Care*. **34**, 76-86 (2020).

257. Widjaja, D.C., Chavez, G.S., Tayko, P.R.M. & Preudhikulpradab, S. Transformational Leadership, Workplace Spirituality And Organizational Performance In A Restaurant Group In Indonesia: A Study Of The Effectiveness Of Organization Development Interventions. *ABAC J.* **40**, 33-57 (2020).

258. Paola, G.*, et al*. Job satisfaction in a sample of nurses: A multilevel focus on work team variability about the head nurse's transformational leadership. *Electronic Journal of Applied Statistical Analysis*. **13**, 713-738 (2020).

259. Chamtitigul, N. & Li, W. The influence of ethical leadership and team learning on team performance in software development projects. *Team Performance Management*. **27**, 240-259 (2020).

260. Wang, Z., Ren, S., Chadee, D., Liu, M. & Cai, S. Team reflexivity and employee innovative behavior: the mediating role of knowledge sharing and moderating role of leadership. *Journal of Knowledge Management*. **25**, 1619-1639 (2020).

261. Ye, Z., Liu, H., Gu, J. & Yang, J. Is relationship conflict totally detrimental to team creativity?: Mediating role of team learning and moderating role of transformational leadership. *Current Psychology*. (2020).

262. Bilal, A., Siddiquei, A., Asadullah, M.A., Awan, H.M. & Asmi, F. Servant leadership: a new perspective to explore project leadership and team effectiveness. *International Journal of Organizational Analysis*. **29**, 699-715 (2020).

263. Jahanshahi, A.A., Maghsoudi, T. & Babaei, F.G.A. What makes teams more innovative in small high-technology ventures? The role of leadership. *International Journal of Entrepreneurial Venturing*. **12**, 251-272 (2020).

264. Ben Sedrine, S., Bouderbala, A. & Nasraoui, H. Leadership style effect on virtual team efficiency: trust, operational cohesion and media richness roles. *Journal of Management Development*. **40**, 365-388 (2020).

265. Torasa, C. & Mekhum, W. The impact of knowledge sharing, human resource management team efficacy and performance on the financial performance: Mediating role of leadership empowerment. *Syst. Rev. Pharm.* **11**, 389-397 (2020).

266. Burmeister, A., Li, Y., Wang, M., Shi, J. & Jin, Y. Team knowledge exchange: How and when does transformational leadership have an effect? *Journal of Organizational Behavior*. **41**, 17-31 (2020).

267. Tran, T.B.H. & Vu, A.D. TRANSFORMATIONAL LEADERSHIP VERSUS SHARED LEADERSHIP FOR TEAM EFFECTIVENESS. *Asian Academy of Management Journal*. **26**, 143-171 (2021).

268. Richardson, T.O., Coti, A., Stroeymeyt, N. & Keller, L. Leadership – not followership – determines performance in ant teams. *Communications Biology*. **4**, (2021).

269. Zaman, U., Florez-Perez, L., Khwaja, M.G., Abbasi, S. & Qureshi, M.G. Exploring the critical nexus between authoritarian leadership, project team member's silence and multi-dimensional success in a state-owned mega construction project. *International Journal of Project Management*. **39**, 873-886 (2021).

270. Bormann, K.C. & Diebig, M. Following an Uneven Lead: Trickle-Down Effects of Differentiated Transformational Leadership. *Journal of Management*. **47**, 2105-2134 (2021).

271. Lai, F.Y., Lin, C.C., Lu, S.C. & Chen, H.L. The Role of Team–Member Exchange in Proactive Personality and Employees’ Proactive Behaviors: The Moderating Effect of Transformational Leadership. *J. Leadersh. Organ. Stud.* **28**, 429-443 (2021).

272. Peng, J., Chen, X., Zou, Y. & Nie, Q. Environmentally specific transformational leadership and team pro-environmental behaviors: The roles of pro-environmental goal clarity, pro-environmental harmonious passion, and power distance. *Human Relations*. **74**, 1864-1888 (2021).

273. Mindeguia, R., Aritzeta, A., Garmendia, A. & Aranberri, A. The Positive Loop at Work: A Longitudinal Long-Term Study of Transformational Leadership, Group Passion, and Employee Results. *Front. Psychol.* **12**, (2021).

274. Duan, Y.*, et al*. Multilevel models of transformational leadership, behavioral integration of top management team and manager ambidexterity in SMEs. *Chinese Management Studies*. **15**, 1009-1031 (2021).

275. Hou, L., Song, L.J., Zheng, G. & Lyu, B. Linking Identity Leadership and Team Performance: The Role of Group-Based Pride and Leader Political Skill. *Front. Psychol.* **12**, (2021).

276. D'Innocenzo, L., Kukenberger, M., Farro, A.C. & Griffith, J.A. Shared leadership performance relationship trajectories as a function of team interventions and members' collective personalities. *Leadership Quarterly*. **32**, (2021).

277. Ali, H.*, et al*. Transformational Leadership and Project Success: Serial Mediation of Team-Building and Teamwork. *Front. Psychol.* **12**, (2021).

278. Vaughan, R.*, et al*. The Rockefeller Team Science Leadership training program: Curriculum, standardized assessment of competencies, and impact of returning assessments. *Journal of Clinical and Translational Science*. **5**, (2021).

279. Ali, A., Wang, H., Bodla, A.A. & Bahadur, W. A moderated mediation model linking transactive memory system and social media with shared leadership and team innovation. *Scand. J. Psychol.* **62**, 625-637 (2021).

280. Van Dijk, D., Kark, R., Matta, F. & Johnson, R.E. Collective aspirations: collective regulatory focus as a mediator between transformational and transactional leadership and team creativity. *J. Bus. Psychol.* **36**, 633-658 (2021).

281. Lisak, A. & Harush, R. Global and local identities on the balance scale: Predicting transformational leadership and effectiveness in multicultural teams. *PLoS One*. **16**, (2021).

282. Imam, H. & Zaheer, M.K. Shared leadership and project success: The roles of knowledge sharing, cohesion and trust in the team. *International Journal of Project Management*. **39**, 463-473 (2021).

283. Chiang, J.T.J., Chen, X.P., Liu, H., Akutsu, S. & Wang, Z. We have emotions but can’t show them! Authoritarian leadership, emotion suppression climate, and team performance. *Human Relations*. **74**, 1082-1111 (2021).

284. He, H. & Hu, Y. The dynamic impacts of shared leadership and the transactive memory system on team performance: A longitudinal study. *Journal of Business Research*. **130**, 14-26 (2021).

285. Han, Z., Ren, H., Yang, S. & Han, Y. Human resource practice management for knowledge intensive team: Impact on team innovation performance and substitution effect of empowerment leadership. *Sustainability (Switzerland)*. **13**, (2021).

286. Maenhout, G., Billiet, V., Sijmons, M. & Beeckman, D. The effect of repeated high-fidelity in situ simulation-based training on self-efficacy, self-perceived leadership qualities and team performance: A quasi-experimental study in a NICU-setting. *Nurse Educ. Today*. **100**, (2021).

287. Castellano, S., Chandavimol, K., Khelladi, I. & Orhan, M.A. Impact of self-leadership and shared leadership on the performance of virtual r&d teams. *Journal of Business Research*. **128**, 578-586 (2021).

288. Huang, T.Y. & Lin, C.P. Is Paternalistic Leadership a Double-Edged Sword for Team Performance? The Mediation of Team Identification and Emotional Exhaustion. *J. Leadersh. Organ. Stud.* **28**, 207-220 (2021).

289. Ali, M., Li, Z., Khan, S., Shah, S.J. & Ullah, R. Linking humble leadership and project success: the moderating role of top management support with mediation of team-building. *International Journal of Managing Projects in Business*. **14**, 545-562 (2021).

290. Eseryel, U.Y., Crowston, K. & Heckman, R. Functional and Visionary Leadership in Self-Managing Virtual Teams. *Group Organ. Manage.* **46**, 424-460 (2021).

291. Lu, H. & Li, F. The Dual Effect of Transformational Leadership on Individual- and Team-Level Performance: The Mediational Roles of Motivational Processes. *Front. Psychol.* **12**, (2021).

292. Chiu, C.Y., Lin, H.C. & Ostroff, C. Fostering team learning orientation magnitude and strength: Roles of transformational leadership, team personality heterogeneity, and behavioural integration. *Journal of Occupational and Organizational Psychology*. **94**, 187-216 (2021).

293. Lorinkova, N.M. & Bartol, K.M. Shared leadership development and team performance: A new look at the dynamics of shared leadership. *Pers. Psychol.* **74**, 77-107 (2021).

294. Lemoine, G.J. & Blum, T.C. Servant leadership, leader gender, and team gender role: Testing a female advantage in a cascading model of performance. *Pers. Psychol.* **74**, 3-28 (2021).

295. Chung, D.S. & Li, J.M. Curvilinear effect of transformational leadership on innovative behavior among R&D teams in South Korea: Moderating role of team learning. *Journal of Organizational Change Management*. **34**, 252-270 (2021).

296. Wu, Q. & Cormican, K. Shared Leadership and Team Effectiveness: An Investigation of Whether and When in Engineering Design Teams. *Front. Psychol.* **11**, (2021).

297. Aubé, C., Francoeur, C., Sponem, S. & Séguin, M. Ethical Leadership and Corporate Board Effectiveness: The Role of Team Reflexivity and Environmental Dynamism. *Group Dynamics*. **25**, 288-302 (2021).

298. Xu, N., Ghahremani, H., Lemoine, G.J. & Tesluk, P.E. Emergence of shared leadership networks in teams: An adaptive process perspective. *Leadership Quarterly*. (2021).

299. Prabhu, N. & Koodamara, N.K. Relationship among transformational leadership, workplace spirituality and team effectiveness: A conceptual framework. *Int. J. Public Sect. Perform. Manage.* **8**, 346-360 (2021).

300. Dahlan, M., Al-Atwi, A.A., Alshaibani, E., Bakir, A. & Maher, K. Diverse group effectiveness: co-occurrence of task and relationship conflict, and transformational leadership. *International Journal of Productivity and Performance Management*. (2021).

301. Rico, R., Uitdewilligen, S.G. & Dorta, D. Patterns of team adaptation: The effects of behavioural interaction patterns on team adaptation and the antecedent effect of empowering versus directive leadership. *J. Contingencies Crisis Manage.* (2021).

302. Ali, A., Wang, H. & Boekhorst, J.A. A moderated mediation examination of shared leadership and team creativity: a social information processing perspective. *Asia Pacific Journal of Management*. (2021).

303. Çakıroğlu, S.S., Caetano, A. & Costa, P. Shared leadership, self-management and perceived team effectiveness in the military context. *Mil. Psychol.* **33**, 308-319 (2021).

304. Hadi, N.U. & Chaudhary, A. Impact of shared leadership on team performance through team reflexivity: examining the moderating role of task complexity. *Team Performance Management*. **27**, 391-405 (2021).

305. Gu, Q., Hu, D. & Hempel, P. Team reward interdependence and team performance: roles of shared leadership and psychological ownership. *Personnel Review*. (2021).

306. Koekemoer, L., de Beer, L.T., Govender, K. & Brouwers, M. Leadership behaviour, team effectiveness, technological flexibility, work engagement and performance during covid-19 lockdown: An exploratory study. *SA Journal of Industrial Psychology*. **47**, (2021).

307. Lauritzen, H.H., Grøn, C.H. & Kjeldsen, A.M. Leadership Matters, But So Do Co-Workers: A Study of the Relative Importance of Transformational Leadership and Team Relations for Employee Outcomes and User Satisfaction. *Review of Public Personnel Administration*. (2021).

308. Joniaková, Z., Jankelová, N., Blštáková, J. & Némethová, I. Cognitive diversity as the quality of leadership in crisis: Team performance in health service during the covid-19 pandemic. *Healthcare (Basel)*. **9**, (2021).

309. Brown, S.G., Hill, N.S. & Lorinkova, N.N.M. Leadership and virtual team performance: A meta-analytic investigation. *Eur. J. Work Org. Psychol.* **30**, 672-685 (2021).

310. Agbonifo, J. Nonstate Armed Groups, Leadership, and Sanctions Effectiveness. *African Security*. **14**, 27-54 (2021).

311. McClean, S.T., Yim, J., Courtright, S.H. & Dunford, B.B. Transformed by the Family: An Episodic, Attachment Theory Perspective on Family–Work Enrichment and Transformational Leadership. *J. Appl. Psychol.* **106**, 1848-1866 (2021).

312. Huang, D., Childs, E., Uppalapati, A.V., Tai, E.C. & Hirsch, A.E. Medical Student Leadership in the Student Oncology Society: Evaluation of a Student-Run Interest Group. *J. Cancer Educ.* (2021).

313. Elyousfi, F., Anand, A. & Dalmasso, A. Impact of e-leadership and team dynamics on virtual team performance in a public organization. *International Journal of Public Sector Management*. **34**, 508-528 (2021).

314. Prabhu, N., Ramaprasad, B.S., Prasad, K. & Modem, R. Does workplace spirituality influence reflexivity in ongoing teams? Examining the impact of shared transformational leadership on team performance. *South Asian Journal of Business Studies*. (2021).

315. Han, J., Yoon, J., Choi, W. & Hong, G. The effects of shared leadership on team performance. *Leadersh. Organ. Dev. J.* **42**, 593-605 (2021).

316. Kabore, S.E., Sane, S. & Abo, P. Transformational leadership and success of international development projects (ID projects): moderating role of the project team size. *Leadersh. Organ. Dev. J.* **42**, 517-530 (2021).

317. Kim, S. & Ishikawa, J. Employee voice mechanisms, transformational leadership, group prototypicality, and voice behaviour: a comparison of portfolio career workers in Japan, Korea and China. *Asia Pacific Business Review*. **27**, 111-144 (2021).

318. Graham, C. & Daniel, H. Fault Lines in Virtual Team Leadership and Team Performance in Undergraduate Virtual Team Short-Term Projects. *International Journal of e-Collaboration*. **17**, 1-14 (2021).

319. Çop, S., Olorunsola, V.O. & Alola, U.V. Achieving environmental sustainability through green transformational leadership policy: Can green team resilience help? *Business Strategy and the Environment*. **30**, 671-682 (2021).

320. Kershaw, C., Rast, D.E., III, Hogg, M.A. & van Knippenberg, D. Divided groups need leadership: A study of the effectiveness of collective identity, dual identity, and intergroup relational identity rhetoric. *J. Appl. Soc. Psychol.* **51**, 53-62 (2021).

321. Mitchell, R. & Boyle, B. Too many cooks in the kitchen? The contingent curvilinear effect of shared leadership on multidisciplinary healthcare team innovation. *Human Resource Management Journal*. **31**, 358-374 (2021).

322. Lyubykh, Z., Gulseren, D., Turner, N., Barling, J. & Seifert, M. Shared transformational leadership and safety behaviours of employees, leaders, and teams: A multilevel investigation. *Journal of Occupational and Organizational Psychology*. **95**, 431-458 (2022).

323. Shoukat, M.H., Elgammal, I., Shah, S.A. & Shaukat, H. Nexus between shared leadership, workplace bullying, team learning, job insecurity and team performance in health care. *Team Performance Management*. **28**, 125-144 (2022).

324. Siswanto & Yuliana, I. Linking transformational leadership with job satisfaction: the mediating roles of trust and team cohesiveness. *Journal of Management Development*. **41**, 94-117 (2022).

325. Akkaya, B. & Bagieńska, A. The Role of Agile Women Leadership in Achieving Team Effectiveness through Interpersonal Trust for Business Agility. *Sustainability (Switzerland)*. **14**, (2022).

326. Marques-Quinteiro, P.*, et al*. A Model of Leadership Transitions in Teams. *Group Organ. Manage.* **47**, 342-372 (2022).

327. Mariam, S., Khawaja, K.F., Qaisar, M.N. & Ahmad, F. Knowledge-Oriented Leadership, Team Cohesion, and Project Success: A Conditional Mechanism. *Project Management Journal*. **53**, 128-145 (2022).

328. Lin, M., Zhang, X., Ng, B.C.S. & Zhong, L. The dual influences of team cooperative and competitive orientations on the relationship between empowering leadership and team innovative behaviors. *International Journal of Hospitality Management*. **102**, (2022).

329. Salanova, M., Rodríguez-Sánchez, A.M. & Nielsen, K. The impact of group efficacy beliefs and transformational leadership on followers’ self-efficacy: a multilevel-longitudinal study. *Current Psychology*. **41**, 2024-2033 (2022).

330. Liu, H.*, et al*. Shared leadership and innovative behavior in scientific research teams: a dual psychological perspective. *Chinese Management Studies*. **16**, 466-492 (2022).

331. Nauman, S., Musawir, A.U., Munir, H. & Rasheed, I. Enhancing the impact of transformational leadership and team-building on project success: the moderating role of empowerment climate. *International Journal of Managing Projects in Business*. **15**, 423-447 (2022).

332. Milley, P. & Szijarto, B. Understanding social innovation leadership in universities: empirical insights from a group concept mapping study. *European Journal of Innovation Management*. **25**, 365-389 (2022).

333. Lisak, A., Harush, R., Icekson, T. & Harel, S. Team Interdependence as a Substitute for Empowering Leadership Contribution to Team Meaningfulness and Performance. *Front. Psychol.* **13**, (2022).

334. Prabhu, N. & Koodamara, N.K. Exploring Shared Leadership: Mediating Roles of Spirit at Work and Team Trust. *Prabandhan Ind. J. Manag.* **15**, 24-39 (2022).

335. Nauman, S., Bhatti, S.H., Imam, H. & Khan, M.S. How Servant Leadership Drives Project Team Performance Through Collaborative Culture and Knowledge Sharing. *Project Management Journal*. **53**, 17-32 (2022).

336. Siangchokyoo, N. & Klinger, R.L. Shared Leadership and Team Performance: The Joint Effect of Team Dispositional Composition and Collective Identification. *Group Organ. Manage.* **47**, 109-140 (2022).

337. Martin, S.R., Emich, K.J., McClean, E.J. & Woodruff, C.T. Keeping Teams Together: How Ethical Leadership Moderates the Effects of Performance on Team Efficacy and Social Integration. *Journal of Business Ethics*. **176**, 127-139 (2022).

338. Yoo, S., JooI, B.K. & Noh, J.H. Team emergent states and team effectiveness: the roles of inclusive leadership and knowledge sharing. *J. Organ. Eff. People Perform.* (2022).

339. Prabhu, N. & Modem, R. Shared transformational leadership and emergent team processes: determinants of team viability and team satisfaction. *Journal of Asia Business Studies*. (2022).

340. Zaman, U., Florez-Perez, L., Anjam, M., Ghani Khwaja, M. & Ul-Huda, N. At the end of the world, turn left: examining toxic leadership, team silence and success in mega construction projects. *Eng. Constr. Archit. Manage.* (2022).

341. Stollberger, J., Ali Al-Atwi, A. & De Cremer, D. Untangling the team social capital–team innovation link: The role of proportional task conflict as well as group- and differentiated individual-focused transformational leadership. *Human Relations*. (2022).

342. Krompa, G.M., O’Mahony, E., Tan, J., Mulligan, O. & Adamis, D. The Effectiveness of Community Mental Health Teams in Relation to Team Cohesion, Authentic Leadership and Size of the Team: A study in the North West of Ireland. *Community Ment. Health J.* (2022).

343. Lungeanu, A., DeChurch, L.A. & Contractor, N.S. Leading teams over time through space: Computational experiments on leadership network archetypes. *Leadership Quarterly*. (2022).

344. Lin, C.P., Liu, C.M. & Hsiao, C.Y. Assessing transactive memory system and team performance: the moderating role of leadership efficacy. *Total Qual. Manage. Bus. Excellence*. **33**, 683-699 (2022).
