# Supplementary material for: Competencies supporting high-performance translational teams: A review of the SciTS evidence base
Source: J Clin Transl Sci. 2023 Mar 3;7(1):e62. doi: 10.1017/cts.2023.17 (PMC10052558; doi:10.1017/cts.2023.17)
Supplement: Supplementary file 1 [file ctssup.zip › S2059866123000171sup003.docx]

**Scoping review protocol:**

(Following JBI Manual for Evidence Synthesis: https://doi.org/10.46658/JBIMES-20-12)

**Title:** A scoping review of evidence for team-emergent competencies associated with team performance

**Introduction:** Although 5 broad competency domains for translational teams were proposed, the specific competencies associated with these domains of affect, communication, management, collaborative problem solving and leadership are not known.

**Definitions**

- Translational Team (TT) -an interdisciplinary team that seeks to improve human health by developing a device, intervention, drug, diagnostic .
- Competencies: knowledge, skills and attitudes demonstrated by a team.
- Team-emergent competencies: KSAs arising from team interactions.
- Performance-advancement across the translational research spectrum.
- Affect-a domain describing that the bonds between TT members are grounded in a concern, empathy and shared regard for others.
- Communication- a KSA where team members effectively exchanges information and integrates team member expertise to solve research problems.
- Management - actions that effectively organize and sustain components of multicomponent investigation.
- Collaborative problem solving- refers to situation where cognitive and social skills of the TT are used to integrate research findings and discipline-grounded interpretations into a cohesive model.
- Leadership- the process providing or supporting the cognitive, resource and affective needs for a TT.

**Details of preliminary searches-**

1. Lotrecchiano GR, Individual and team competencies in translational teams.J Clin Transl Sci. 2020 Oct 21;5(1):e72. doi: 10.1017/cts.2020.551.

A modified Delphi process consisting of (1) a literature review, (2) an initial list of competencies, (3) successive rounds of review and analysis, and (4) iterative edits resulted in consensus among the authors for a definition of translational science teams and a competency framework inclusive of individual and team components.

24 manuscripts were cited in the paper. These were drawn from a literature review that identified 500 papers, based on the following search strategy: (“Translational Medical Research”[Mesh] OR “translational research” OR “translational science” OR “translational medical research” OR translational[tiab] OR transl[All Fields] OR (translational[tiab] AND (scientist* OR researcher*))) AND (team OR teams OR teamwork OR collaboration OR cooperative OR cooperation OR “Intersectoral Collaboration”[Mesh] OR “Interprofessional Relations”[Mesh]) AND (skill OR skills OR competency OR competencies OR competence OR “Professional Competence”[Mesh] OR “Competency-Based Education”[Mesh]) NOT (animals[mh] NOT (humans[mh] AND animals[mh])). Group competency-relevant literature was identified using the strategy: (“Translational Medical Research”[Mesh] OR “translational research”[tiab] OR “translational science”[tiab]OR “translational medical research”[tiab] OR translational[tiab] OR transl [All Fields]) AND (team OR teams OR teamwork OR “Intersectoral Collaboration”[Mesh] OR “Interprofessional Relations”[Mesh]) AND (“Cooperative Behavior”[Mesh] OR “Group Processes”[Mesh] OR “group processes” OR “group process” OR “group behavior” OR “group behaviors” OR “group behaviour” OR “group behaviours” OR “cooperative behavior” OR “cooperative process” OR “cooperative processes” OR “group processes” OR “group process” OR “team communication” OR “team mentoring”).

1. Calhoun, WJ. The CTSA as an exemplar framework for developing multidisciplinary translational teams. Clin Transl Sci. 2013 Feb;6(1):60-71. doi: 10.1111/cts.12004. Epub 2012 Oct 17.

This paper is a survey of organizational team types from business and management literature with 71 primary references. An annotated bibliography of 198 references was developed and available as Supplementary Information. This bibliography was divided into six Sections: Section 1, General Reviews, Effectiveness Studies, Meta-Analyses; Section 2 Team Development, Evolution, Teamwork Behavior and Skills; Section 3 Science of Team Science; Section 4 Disciplinarity, Collaboration; Section 5 Reviews, Critiques of Translational Research; and Section 6, Innovation, Knowledge Management, R&D Team Management, Product Development, Cross-Functional Team.

**Overall review objective**- Identify what specific behaviors or skills in these competency domains most directly impact TT performance?

**Search strategy used for this review:**

A comprehensive literature review was conducted in the Medline Core Collection to update literature identified in prior reviews from 2010-2022, resulting in 344 manuscripts.

Data Bases included: Current Contents Connect, Medline and Scopus Data bases.

Search strategy: ((TI=((Leadership) AND (team OR teams OR group OR groups) AND (success* OR failure* OR performance OR effective* OR innovat* OR transform* OR evaluat* OR assess* OR functional OR antecedent* OR measure* OR model OR models))) OR ((TS=((leadership) Near/7 (team OR teams OR group OR groups) Near/7 (success* OR failure* OR performance OR effective* OR innovat* OR transform* OR evaluat* OR assess* OR functional OR antecedent* OR measure* OR model OR models))) AND (SO = (JOURNAL OF CLINICAL "AND" TRANSLATIONAL SCIENCE OR JOURNAL OF MANAGEMENT OR GROUP ORGANIZATION MANAGEMENT OR GROUP ORGANIZATION STUDIES OR JOURNAL OF APPLIED PSYCHOLOGY OR LEADERSHIP QUARTERLY OR ORGANIZATIONAL DYNAMICS OR ACADEMY OF MANAGEMENT JOURNAL OR EVALUATION THE HEALTH PROFESSIONS)))) AND (PY=(2010-2023)) AND (DT=("ARTICLE" OR "REVIEW")) AND (LA=(English)) AND (SILOID=("WOS" OR "CCC" OR "MEDLINE")).

**Eligibility criteria for inclusion into the analysis:**  344 manuscripts identified in the literature search from 2010-2022. These were combined with the two prior comprehensive reviews on Translational Teams cited above to produce 488 candidate mansucripts (Figure 1, below).

Abstracts were scanned – articles that met Performance as outcome, studied a relevant team competency domain and included relevant team types were retained. These criteria were:

1. Team competency domain in study outcome. 101 abstracts were excluded that did not address a competency related to the 5 domains. .
2. Team-type relevance: 241 abstracts did not study relevant teams- such as knowledge-generating, product development, innovation, translational team. Excluded were virtual teams, business teams, service industry teams, etc.
3. Performance as an ouitcome. 60 excluded were studies that looked at individual satisfaction, organizational impact, etc.

*Note that the number of exclusions exceed the number of manuscripts since several manuscripts were not related to competency domain nor studied performance.

488 candidate manuscripts

344 manuscripts

Categorized for competency domain

cited in manuscript

Relevance screen

Literature search 2010-present

Lotrecchiano et al

Calhoun et al

101 not related to competency domain

241 unrelated to translational teams

60 Performance not an outcome

162 core manuscripts

**Selection and classification of articles.**  162 core papers were retained for content of this review.

A classification table for data extraction of the core papers is shown -**Supplemental Table 3**. For each paper, the citation number, title, relevance to competency domain (Affect, Comm, communication; Mgmt, management; CPS, collaborative problem solving; Leader, leadership), description of an intervention (individual or team-based), and perceived relevance is scored. The perceived relevance is a qualitative estimation made by the authors based on the size of the study (number of groups) and relevance to academic TTs (High, Hi, Medium, Med, Low, Lo). Notes are added for clarification of scoring, or unusual characteristics that may make generalizability uncertain, such as geographical focus.

**Conflict resolution-** negotiation among authors

**How data will be presented** – Papers are grouped by primary relevance to the 5 major competency domains, and summarized in text.
